# Granulomatous Diseases of the CNS: Aetiology, Neuroradiological Features and Clinical Findings

**DOI:** 10.1007/s00062-026-01672-2

**Published:** 2026-06-02

**Authors:** Stefan Weidauer, Randolf Klingebiel

**Affiliations:** 1 Institute of Neuroradiology, Goethe University Frankfurt, Frankfurt am Main, Germany; 2 Clinic of Diagnostic and Interventional Radiology / Neuroradiology, Sankt Elisabeth Hospital, Gütersloh, Germany

**Keywords:** Granulomatous disease, Central nervous system, MRI, Vasculitis, Autoimmune, Infectious

## Abstract

Granulomatous disorders of the central nervous system (CNS) encompass a highly heterogeneous group of diseases with autoinflammatory, autoimmune, infectious, proliferative-neoplastic, foreign body-related, or drug-induced aetiologies. Neuroimaging plays a pivotal role, particularly with respect to early diagnosis. Therefore, knowledge of specific imaging patterns is crucial in order to narrow down the broad differential diagnosis. Although imaging cannot replace histology, its importance in initiating therapy as early as possible is indisputable. This is particularly true since many granulomatous diseases respond to treatment, usually with immunosuppressants and/or anti-infectives. This review highlights representative disease entities across the major aetiological categories and discusses key neuroradiological features and clinical symptoms together with relevant differential diagnostic considerations.

## Introduction

Granulomatous inflammatory diseases of the central nervous system (CNS) comprise a broad and diverse spectrum of underlying aetiologies, including autoimmune, infectious, and proliferative-neoplastic disorders [1–3]. In addition, granulomatous reactions may occur secondary to exposure to foreign material or in the context of immunodeficiency (Table 1; [3]). Histopathologically, granulomas represent a chronic inflammatory response to persistent antigenic stimulation, characterized by the accumulation of macrophages and activated T lymphocytes (Th1, Th2, and Th17 subsets). In addition to infectious agents, autoantigens may function as the inciting “foreign” stimulus [2, 3].

**Table 1:** Granulomatous CNS diseases (selection)

	Etiology	Diseases/provoking factors
1	Autoinflammatory	Sarcoidosis
		M. Crohn
		Blau syndrome
		Other
2	Autoimmune related	*a) Vasculitis (notably primary vasculitis)*
		Giant cell arteritis
		Primary CNS vasculitis (PCNSV)
		ANCA positive vasculitis: Granulomatosis with polyangiitis (GPA; Wegener’s granulomatosis); Eosinophilic granulomatosis with polyangiitis (EGPA; Churg-Strauss syndrome)
		Amyloid‑β related angiitis (ABRA)
		Cerebral amyloid angiopathy (CAA) related inflammation (CAA—ri)
		Other
		*b) IgG 4‑related diseases (RD)*
		Hypertrophic pachymeningitis
		Orbital involvement
		Hypophysitis, other
		*c) Idiopathic orbital inflammation (IOI)*
		Tolosa-Hunt syndrome (THS), other
		*d) Other*
3	Infectious	*a) Bacterial*
		Mykobacterium (e.g., M. tuberculosis)
		Treponema (e.g., T. pallidum/syphilis), other
		*b) Parasitic*
		Toxoplasmosis
		Schistosoma
		Leishmania
		Amoeba
		Toxocariasis canis, other
		*c) Fungal*
		Aspergillus
		Cryptococcus
		Blastomycosis, other
4	Proliferative/neoplasm	*a) Langerhans cell histiocytosis (LCH)*
		Langerhans cell histiocytosis (LCH)
		Erdheim-Chester disease
		Rosai-Dorfman disease
		Juvenile xanthogranuloma
		Histocytic sarcoma
		*b) Lymphomatoid granulomatosis*
		*c) Other*
5	Foreign body	Foreign body reaction to embolic materials in neurointerventional procedures (NICE; non-ischemic cerebral enhancing lesions)
		Cholesterol crystals
		Metal abrasion
		Silicates, beryllium, talcum, other
6	Immune deficiency	Septic granulomatosis
		General variable immune deficiency
7	Drug induced, allergic, other	Sarcoid-like lesions in malignoma
		Checkpoint inhibitors
		Type 1 interferons
		TNF‑α antagonists

Abbreviations: *ANCA* Antineutrophilic cytoplasmatic antibodies, *TNF* Tumor necrosis factor

Several histological granuloma subtypes are recognized, including non-caseating epithelioid granulomas as seen in sarcoidosis, caseating granulomas typical of tuberculosis, necrotizing granulomas, and complex granulomas induced by highly immunogenic antigens and regulated by adaptive immune responses [2–4]. Owing to dense inflammatory cell infiltration, effective phagocytosis is often impeded, resulting instead in lesion demarcation from surrounding tissue [3, 4].

**Fig. 1:**
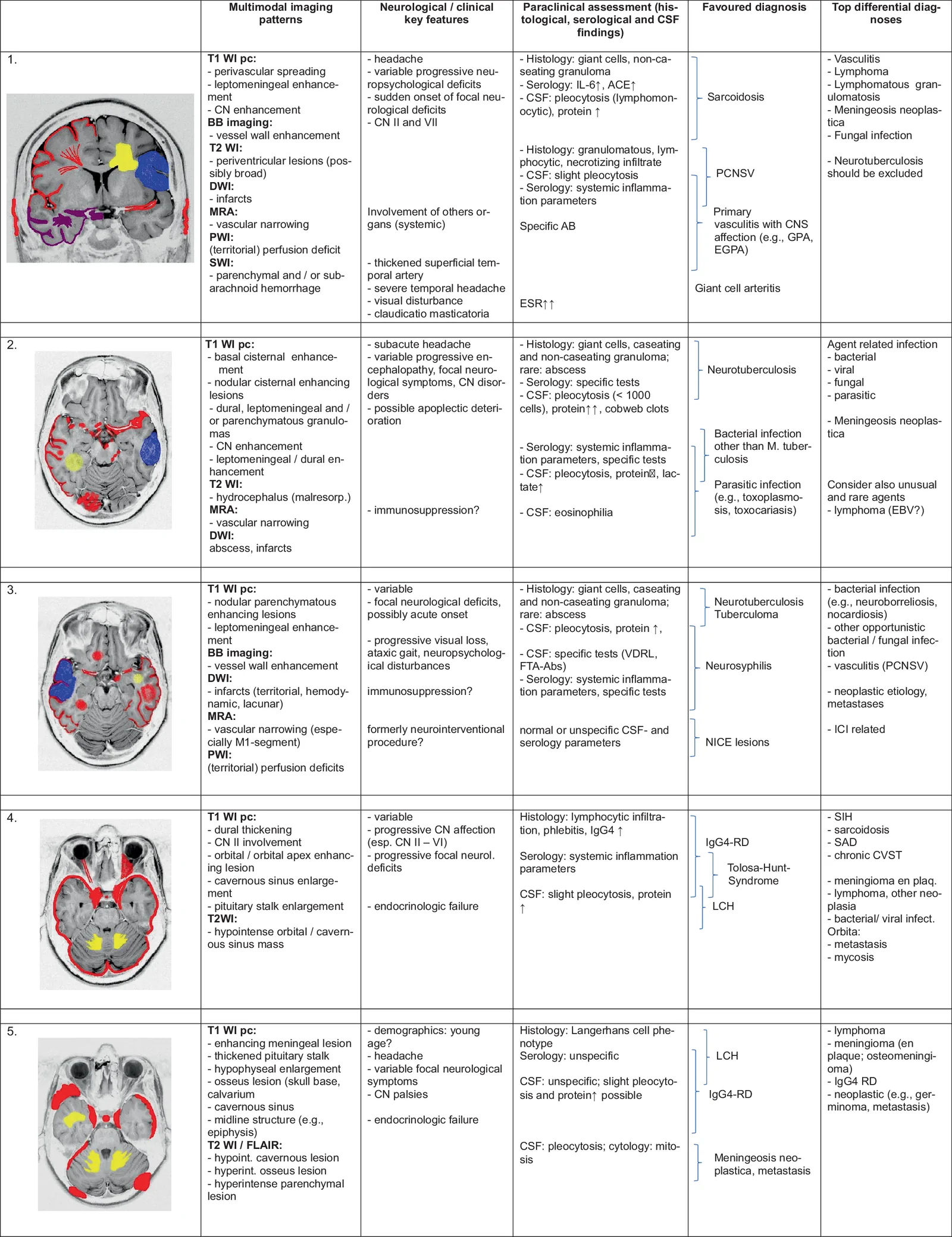
Multimodal imaging pattern and differential diagnostic considerations. Colours: red: T1 enhancing lesions; blue: ischemic lesions; yellow: T2 hyperintense white matter lesions; yellow punctuated: abscess; magenta: bleeding. Abbreviations: *AB* Antibody, *ACE* Angiotensin converting enzyme, *BB* Black blood, *CN* Cranial nerve, *CSF* Cerebrospinal fluid, *CVST* Cerebral vein and/or sinus thrombosis, *DWI* Diffusion weighted imaging, *EBV* Epstein Barr Virus, *EGPA* Eosinophilic granulomatosis with polyangiitis, *ESR* Erythrocyte sedimentation rate, *FLAIR* Fluid attenuated inversion recovery, *FTA*-*Abs* Fluorescent treponemal antibody absorption, *GPA* Granulomatosis with polyangiitis, *ICI* Immune checkpoint inhibitor, *IgG4-RD* Immunoglobulin G4 related disease, *Il‑6* Interleukin‑6, *LCH* Langerhans cell histiocytosis, *MRA* Magnetic resonance angiography, *M.* *tuberculosis* Mycobacterium tuberculosis, *NICE* Non-ischemic cerebral enhancing lesions, *PCNSV* Primary central nervous system vasculitis, *PI* Perfusion imaging, *SAD* Systemic autoimmune disease, *SIH* Spontaneous intracranial hypotension, *VDRL* Venereal disease research laboratory, *WI* Weighted images

A definitive aetiological diagnosis is achieved only in a minority of cases, for example through biopsy-based identification of pathogens or foreign material, detection of epithelioid granulomas suggestive of sarcoidosis [3–6], or demonstration of multinucleated giant cells in granulomatous vasculitis [7–11]. From a neuroradiological perspective, distinguishing granulomatous inflammation from neoplastic disease may be challenging [2, 8]. Consequently, interdisciplinary correlation of neurological, rheumatological, and infectious disease findings, supplemented by targeted serum and cerebrospinal fluid (CSF) analyses, is indispensable in the diagnostic work-up [2, 10, 11]. However, multiparametric MRI provides additional information, thereby partially overcoming the limitations of individual imaging sequences ([12–16]; Fig. 1). Furthermore, quantitative multiparametric imaging offers additional insight into the underlying pathophysiological processes [12, 17]. However, a detailed description including artificial intelligence-based imaging analysis, goes beyond the scope of this review, and reference is made to the relevant further reading [12, 13, 17]. This article summarizes the most relevant neuroradiological and clinical features of prototypical granulomatous CNS disorders and provides practical guidance for differential diagnosis (Fig. 1).

## Autoinflammatory Disorders: Sarcoidosis

Aetiology: Sarcoidosis is a systemic autoinflammatory granulomatous disease of unknown aetiology [3–6, 18]. Histologically, it is characterized by non-caseating epithelioid cell granulomas composed of macrophages and lymphocytes, frequently accompanied by multinucleated giant cells [3, 4]. In contrast to tuberculosis, central necrosis is absent [3]. CNS involvement occurs in approximately 5% of patients (reported range 2–16%) [4, 18–25]. Neurosarcoidosis is categorized as definite, probable, or possible [20, 22, 24]. Alternative diagnoses must be rigorously excluded ([4, 26-28]; Fig. 1). Definite neurosarcoidosis requires histopathological confirmation, whereas probable neurosarcoidosis is diagnosed based on compatible MRI findings and/or laboratory evidence of CNS inflammation [4, 23, 24]. CSF analysis typically reveals lymphocytic or monocytic pleocytosis with elevated protein concentrations. Oligoclonal bands (OCB) in CSF are detected in approximately 70% of cases [20]. Serum biomarkers such as angiotensin-converting enzyme (ACE), soluble interleukin‑2 receptor, and neopterin may support the diagnosis, although they can be normal, particularly in isolated neurosarcoidosis [4, 18–23]. The clinical manifestations and neuroradiological findings of isolated neurosarcoidosis largely mirror those observed in systemic sarcoidosis with CNS involvement (Table 2).

**Table 2:** Neurological findings and neuroimaging features in neurosarcoidosis

*a) Neurologic symptoms*
Cranial nerve (CN) involvement: 63–72%; CN II (often bilateral) > CN VII, IX and III
Spinal symptoms: 19–28%
Aseptic meningitis: 18–26%
Epileptic seizures: 17%
Organic psychosis: 10%
Hypothalamic/pituitary symptoms: 2–3%
Peripherial nervous system involvement
Sceletal muscles
*b) Neuroradiological features on MRI*
Generally: sensitivity approximately 80%, low specifity
Diffuse or focal dural and/or leptomeningeal thickening with (distinct) enhancement on T1w after contrast medium (CM) administration
Infundibular/hypothalamic volume increase and enhancement on T1w after CM administration
Enhancement of CN on T1w after CM administration
Parenchymal lesions: on T2/FLAIR periventricular accentuated, also brainstem and basal ganglia; streaky perivascular enhancement (angiocentric pattern) on T1w after CM administration
Spinal involvement (4 subtypes on T1w after CM administration)
1. Subpial enhancement with dorsal accentuation (trident sign)
2. Nodular radicular, leptomeningeal
3. Pancake-like with extensive hyperintense signal alteration of the spinal cord on T2w (longitudinal extensive transverse myelitis; LETM)
4. Tumefactive; short segment space-occupying

**Fig. 2:**
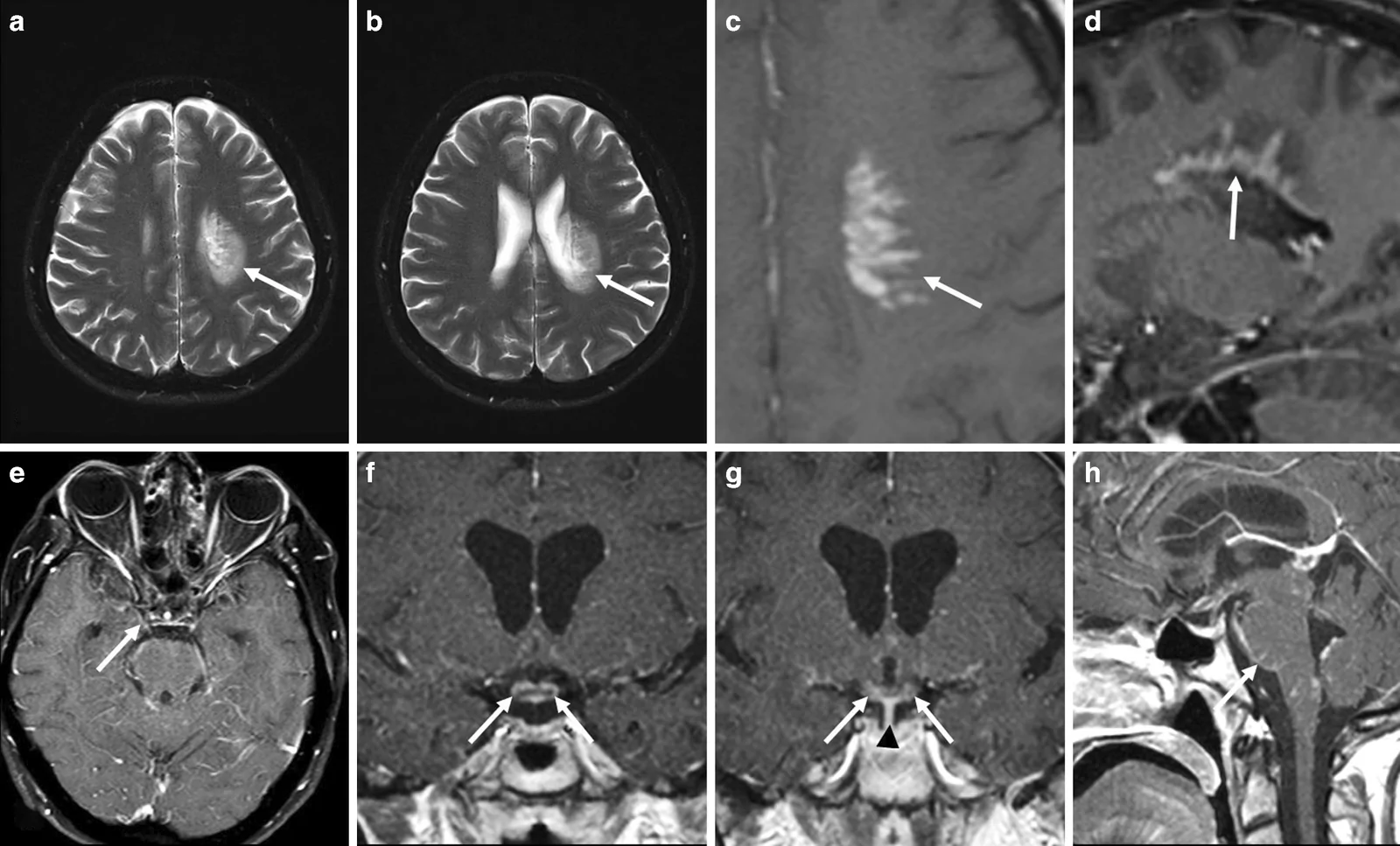
Neurosarcoidosis. **a**, **b** inhomogeneous hyperintense lesion on T2-weighted images (WI) in the left centrum semiovale (arrow) adjacent to the cella media in a 57-year old woman with progressive right sided hemiparesis; **c**, **d** distinct enhancement with angiocentric pattern (arrow) on T1 WI after contrast medium (CM) administration; **e**–**h** T1 WI after CM administration showing enhancement of the cranial nerves (**e**, arrow: oculomotor nerve; **f**, **g** optic nerve and chiasm, arrow), the pituitary stalk (**g**, black arrowhead) and the pontine perivascular spaces (**h** arrow)

**Fig. 3:**
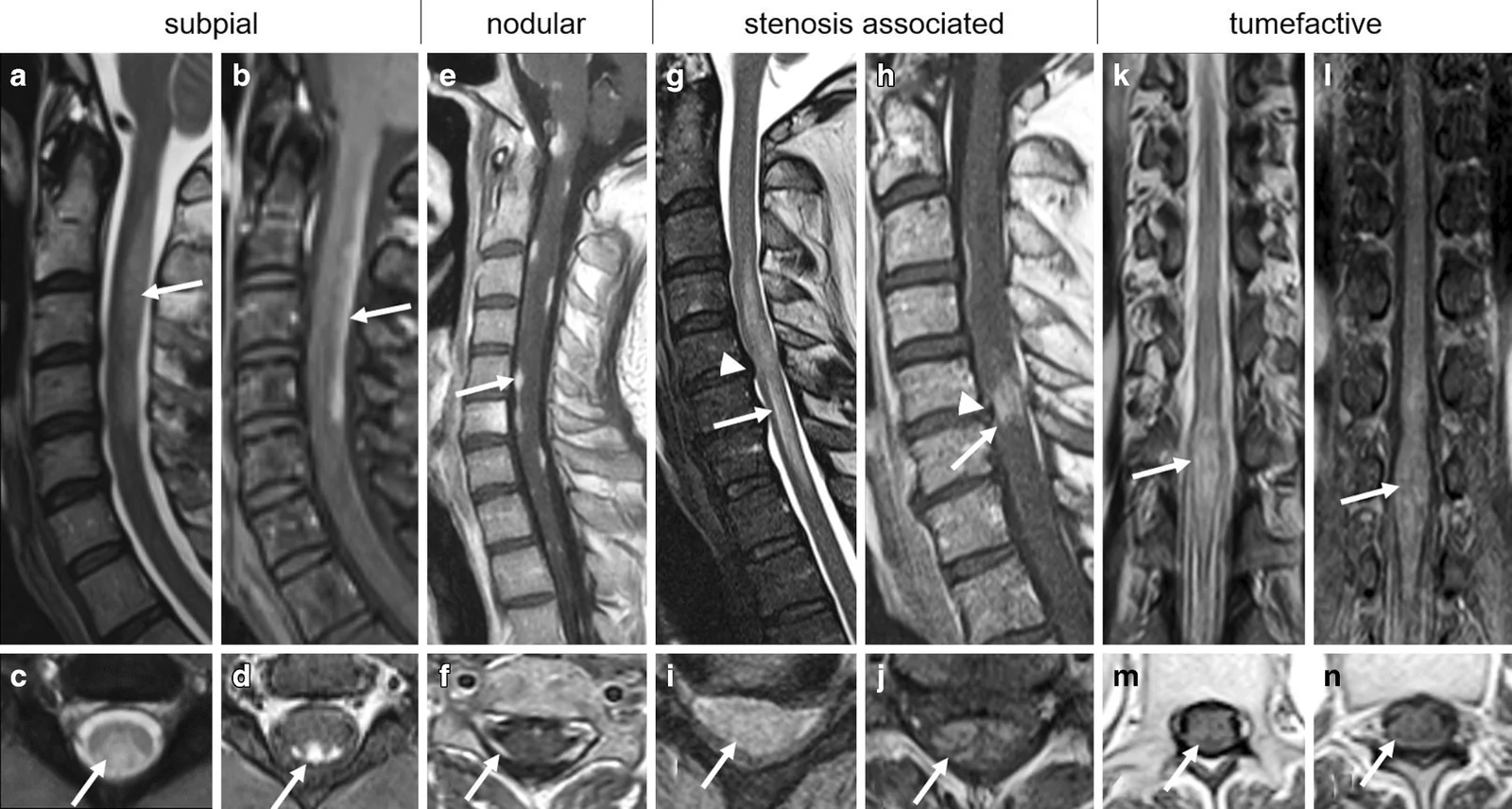
Different lesion patterns in spinal neurosarcoidosis. **a**–**d** subpial type with dorsolateral accentuated contrast enhancement (CE) (**b**, **d** arrow; **d** trident sign) in longitudinal extensive transverse myelitis (LETM; **a**, **c** arrow). **e**, **f** nodular type disclosing multifocal radicular and leptomeningeal enhancement (arrow) on post contrast (pc) T1 WI. **g**–**j** stenosis associated type showing extensive hyperintense signal changes on T2 WI (**g**, **i** arrow) with focal CE (**h**, **j** arrow) and pancake sign (**j** arrow) adjacent to the moderate spinal stenosis (**g**, **h** arrowhead); **k**–**n** tumefactive type with enlargement of the thoracolumbar spinal cord and the conus medullaris (**k**, **l**, **m** arrow) and inhomogeneous intramedullar (**l**, **m** arrow) and radicular (**n** arrow) CE on pc T1 WI

**Fig. 4:**
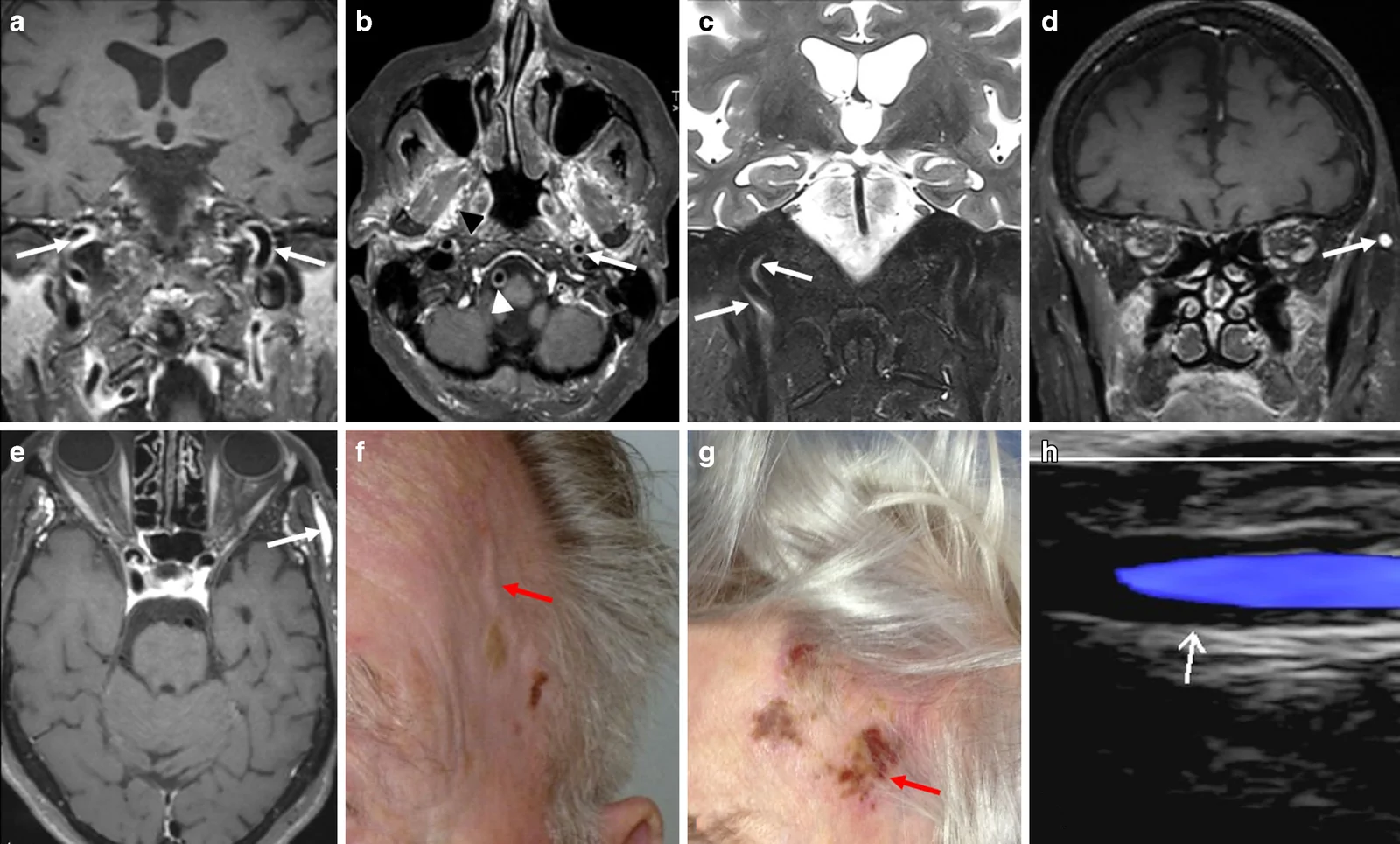
Giant cell arteritis (arteritis cranialis). **a**–**e** Vessel wall enhancement of the internal carotid artery (ICA; **a**, **b** arrow), the basilar artery (**b** white arrowhead) and the superficial temporal artery (**d**, **e** arrow) on post contrast (pc) T1 weighted images (WI); hyperintense signal on T2 WI (**c** arrow); note enhancement of the pterygoid muscles (**b** black arrowhead); **f**–**h** thickened superficial temporal artery (**f** arrow), cutaneous necrosis (**g** arrow) and “halo sign” in Doppler ultrasound (**h** arrow)

Imaging pattern: Characteristic imaging features of cranial neurosarcoidosis include a) enlargement of the infundibulum and hypothalamus, b) periventricular, brainstem, and basal ganglia T2-hyperintense lesions, c) perivascular or angiocentric contrast enhancement, d) cranial nerve enhancement, most commonly involving the optic nerve and e) focal dural or leptomeningeal thickening with enhancement (Fig. 2; [2, 4, 24, 25]). In spinal involvement, four distinct imaging subtypes are recognized, i.e., a) subpial, b) nodular, c) stenosis associated and d) tumefactive (Fig. 3; [29–32]).

## Autoimmune Granulomatous Disorders

The most important autoimmune-mediated diseases associated with granuloma formation are listed in Table 1. In addition to primary vasculitides [7–9, 32] such as giant cell arteritis, primary angiitis of the CNS (PACNS), and ANCA (Antineutrophilic cytoplasmatic antibodies) positive vasculitides, IgG4-related diseases [33–35] and Tolosa-Hunt syndrome [36, 37] are presented in more detail below.

- Vasculitides

In Table 3 vasculitides with CNS involvement according to the revised Chapel Hill Classification (2012) are summarized [32]. Classification is primarily based on the size of affected arteries (large, medium, or predominantly small vessels), rather than on primary or secondary CNS involvement.

**Table 3:** CNS vasculitides (classification after Chapel Hill, revised 2012) [32]

*1. Vasculitides of the central nervous system (CNS) and/or the cervical arteries*
Giant cell arteritis
Takayasu arteritis
Primary CNS vasculitis (PCNSV)
*2. Vasculitides especially of the medium sized vessels*
Polyarteritis nodosa
Kawasaki syndrome
*3. ANCA*—*positive vasculitides especially of the small sized vessels (AAV)*
Granulomatosis with polyangiitis (GPA; Wegener’s granulomatosis)
Eosinophilic granulomatosis with polyangiitis (EGPA; Churg-Strauss syndrome)
Microscopic polyangiitis
*4. Other vasculitides with possible cerebral involvement*
Behçet syndrome
Sjögren syndrome
Systemic lupus erythematodes (SLE)
Antiphospholipid antibody syndrome
Cogan syndrome
IgA related vasculitis
Vasculitides due to infection, malignant diseases, drugs, other

Abbreviations: *ANCA* Antineutrophilic cytoplasmatic antibodies, *AAV* ANCA associated vasculitides

### Giant Cell Arteritis

Giant cell arteritis (GCA) is the most common primary systemic vasculitis, predominantly affecting women over the age of 50 years [32, 38–40]. Approximately 30–40% of patients also suffer from polymyalgia rheumatica, which is substantially more prevalent than GCA. The disease primarily involves the branches of the external carotid artery (ECA), and less frequently the vertebral artery [38–40].

*Temporal (cranial) arteritis:* Clinically, patients present with progressive temporal headaches over days to weeks, reduced general condition, and often bilateral tenderness and prominence of the superficial temporal artery [38–40]. Laboratory findings typically include elevated erythrocyte sedimentation rate (ESR), increased C‑reactive protein (CRP), and leukocytosis. Due to segmental involvement (“skip lesions”), biopsy—if performed—should include at least 10 mm of vessel length [38–40].

Imaging pattern: MRI may demonstrate concentric vessel wall thickening and enhancement on post-contrast T1-weighted images (WI), particularly when using black-blood techniques, i.e. direct MRI signs of arteritis [41, 42]. Hyperintense vessel wall signal on fat-suppressed T2 WI may provide additional evidence in favour of inflammatory involvement (Fig. 4). Duplex ultrasonography shows the characteristic halo sign caused by inflammatory oedematous wall thickening. Involvement of the internal carotid artery at the origin of the ophthalmic artery, or direct involvement of the latter, may result in acute visual loss with potentially irreversible blindness within hours [40, 43]. In such cases, immediate high-dose intravenous corticosteroid therapy (e.g. 500–1000 mg prednisolone per day) is mandatory. Other typical manifestations include jaw claudication and skin necrosis due to involvement of branches of the external carotid artery [38, 39].

**Fig. 5:**
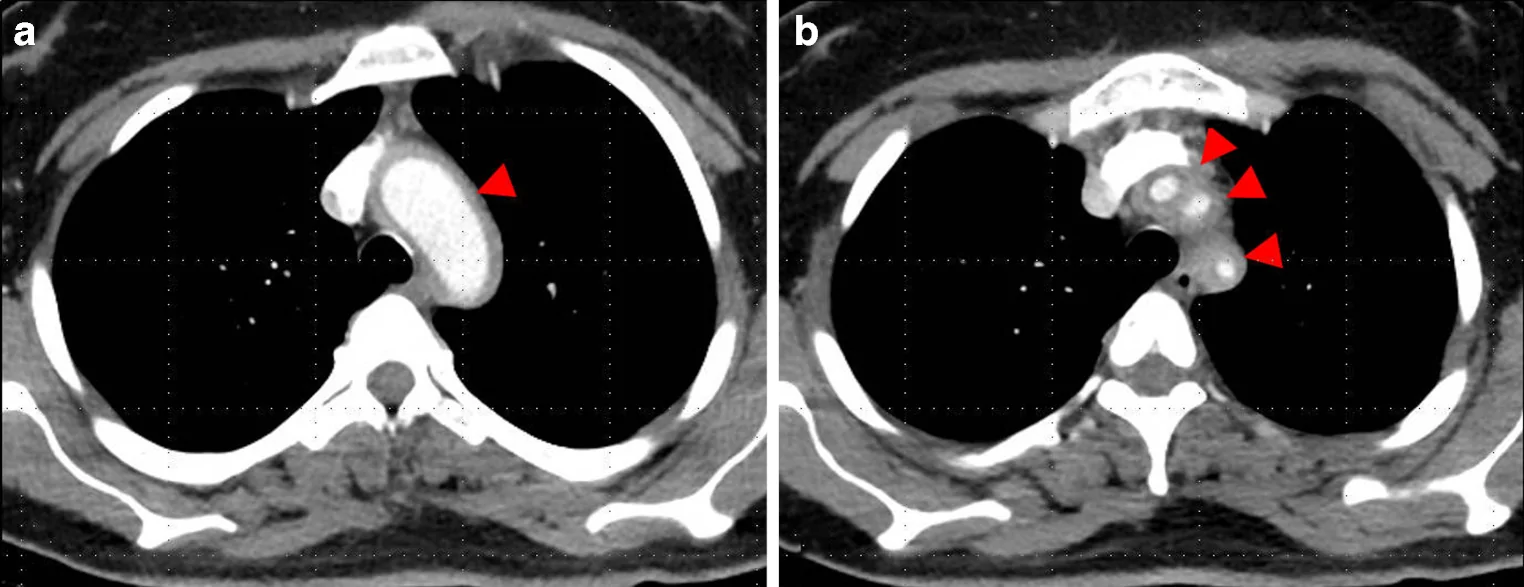
Giant cell arteritis (Takayasu arteritis) in a 34-year old woman suffering from an acute left hemispheric syndrome. **a**, **b** CT angiography showing thickened vessel walls of the aortic arch (**a** arrowhead) and the proximal supraaortal branches (**b** arrowhead)

*Takayasu arteritis: *This subtype of GCA often affects large body vessels, particularly the aorta, especially the aortic arch and the proximal supraaortal branches. It most commonly occurs in women aged under 40 years [38, 39]. In Fig. 5 typical imaging findings in Takayasu arteritis are presented.

### Primary Angiitis of the CNS (PACNS)

Aetiology: PACNS is an autoimmune mediated inflammatory disease of medium-sized and small vessels confined exclusively to the CNS, with unknown aetiology [7–10, 44–46]. Histologically, three subtypes are distinguished: a) granulomatous (58%), often with amyloid‑β deposition, b) lymphocytic (28%), and c) necrotizing (14%), associated with fibrinoid necrosis and haemorrhage [9, 38, 39]. The differentiation from inflammatory variants of cerebral amyloid angiopathy (CAA) is important [47–52]. In amyloid-β-related angiitis (ABRA), transmural inflammation is present, whereas CAA-related inflammation is characterized predominantly by perivascular infiltrates [47, 48]. However, in recent publications, these entities are often grouped together [7, 9].

Key clinical features: Clinical hallmark symptoms include headache, neuropsychological impairment, and/or focal neurological deficits [7–9, 45, 52–56]. There are no specific serum markers. ESR may be elevated, and CRP is increased in < 25% of cases. CSF analysis shows lymphocytic pleocytosis in > 95% during the disease course [7–9, 55]. Diagnosis relies primarily on imaging and positive biopsy findings [7–9, 45, 46, 54–56].

Imaging pattern: MRI may reveal vessel wall enhancement on black blood sequences [41, 42, 46, 55]. In addition, white matter lesions predominantly in the centrum semiovale, infarctions possibly recurrent and in different vascular territories, or haemorrhages (intracerebral or subarachnoid) as indirect signs of vasculitis occur frequently [9, 46, 54, 55]. Conversely, the diagnosis of PACNS is unlikely if both, MRI and digital subtraction angiography (DSA), are unremarkable [9, 46, 55]. However, also spinal cord involvement in PACNS is reported [57, 58].

MRI is highly sensitive (> 90%) but has limited specificity [7–9, 56]. Infarcts are detected in 53%, contrast-enhancing lesions in 33%, and leptomeningeal enhancement in 8% [7–9, 46, 55]. Tumefactive lesions are rare (< 5%) [49].

**Fig. 6:**
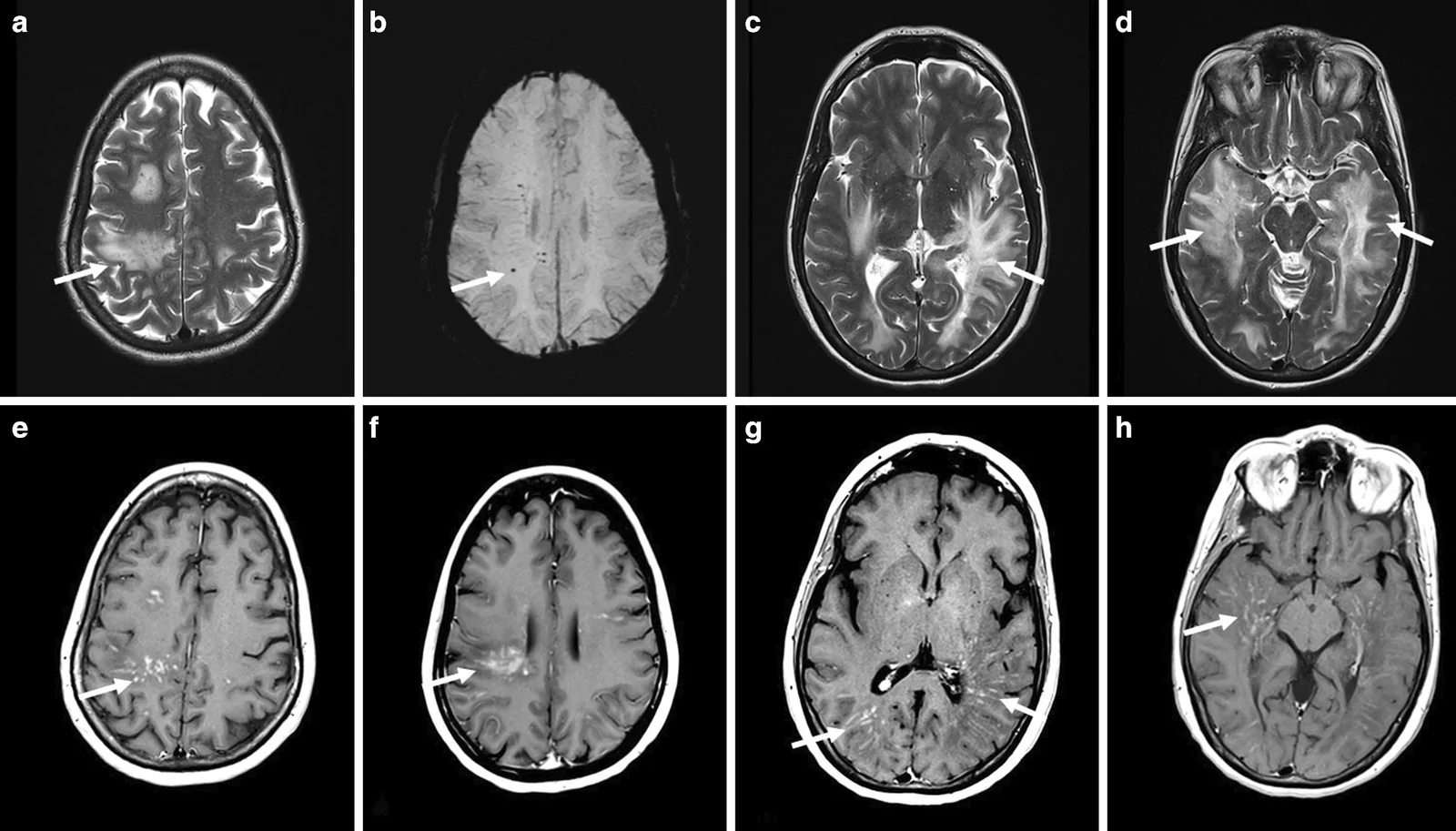
Primary CNS vasculitis in a 52-year old woman suffering from rapid progressive cognitive impairment and dizziness. Biopsy disclosed fibrinoid vessel wall necrosis and lymphocytic infiltration; cerebrospinal fluid (CSF) analysis: 67 cells (lymphocytes, protein content 79 mg/100 ml). **a**–**d** large and inhomogeneous partially symmetric hyperintense lesions on T2 weighted images (WI) (**a**, **c**, **d** arrow) with multifocal punctuate bleedings (**b** arrow); **e**–**h** multifocal contrast enhancement (CE) on post contrast (pc) T1 WI (arrow), partially with angiocentric linear pattern (**g**, **h** arrow)

**Fig. 7:**
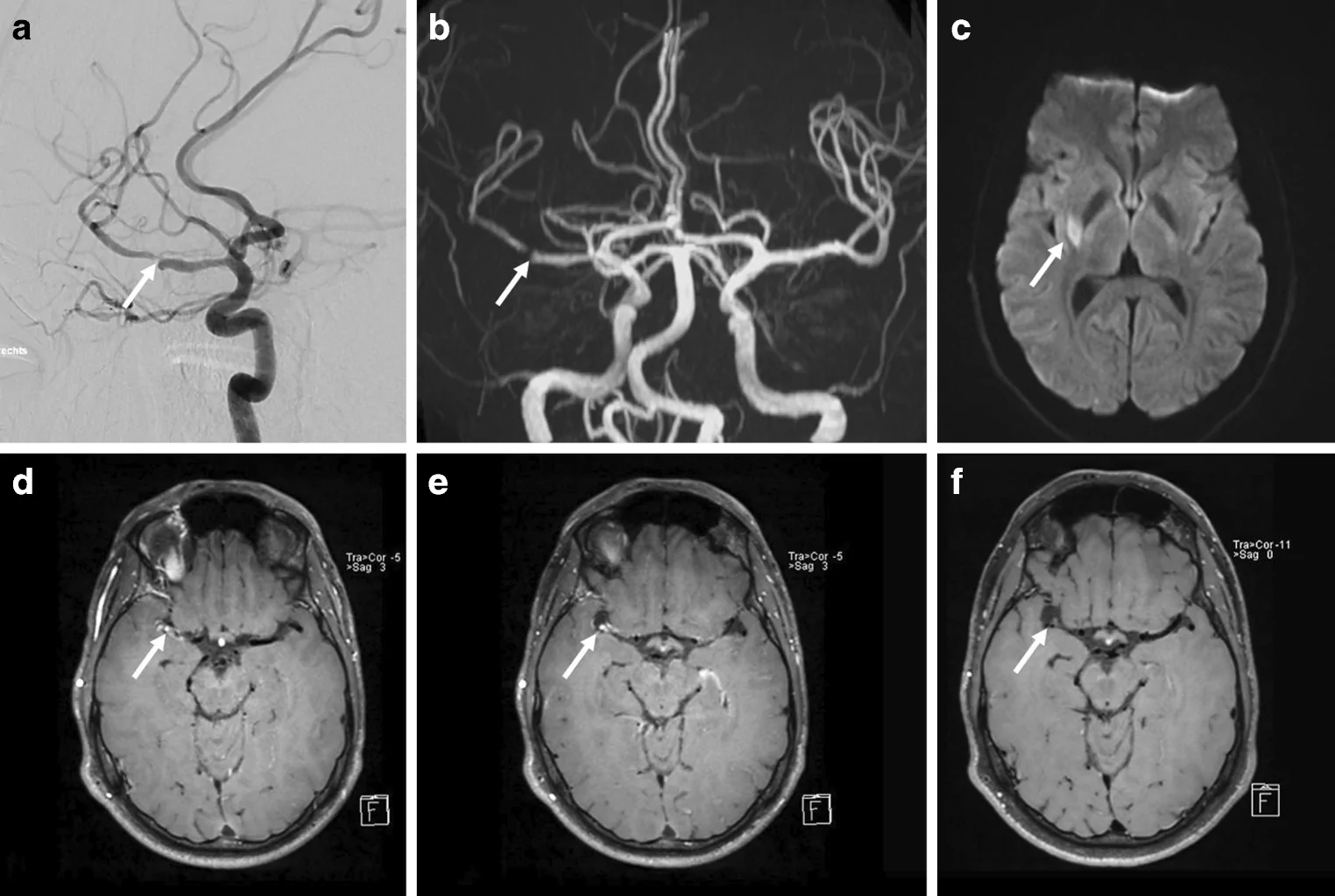
Primary CNS vasculitis in a 23 year old man suffering from acute left-sided hemiparesis; cerebrospinal fluid (CSF) analysis: no pleocytosis, slight elevated protein content. **a**–**c** digital subtraction angiography (DSA) and MR angiography showing severe stenosis in the distal M1-segment right (**a**, **b** arrow); lacunar infarct (**c** arrow) on diffusion weighted imaging (DWI; b = 1000 s/mm^2^) (**c** arrow;); **d**–**f** post contrast T1 WI (black blood sequence) disclosing circumscribed vessel wall enhancement of the right-sided distal M1- and proximal M2-segment (**d**, **e** arrow). Complete resolution of imaging findings is depicted on a follow-up study at ten months following immunomodulatory therapy (**f** arrow)

DSA visualizes only large and medium-sized vessels because small vessels are below the resolution of angiography in a routine setting [7, 8]. Typical angiographic findings such as luminal irregularities or a “string-of-beads” pattern are suggestive but not pathognomonic for vasculitis [7–9, 46, 54, 55]. Differential diagnosis: Differential diagnoses include reversible cerebral vasoconstriction syndrome (RCVS), vasospasm after subarachnoid haemorrhage (SAH), and also metabolic vasculopathies (see also Fig. 1; [7, 10]). The two characteristic PACNS imaging patterns, i.e., predominant involvement of larger vessels and accentuated affection of the small vessels, are summarized in Table 4 (Figs. 6 and 7).

**Table 4:** Typical paraclinical findings in primary CNS vasculitis (PCNSV)

	Medium sized vessels (70%)	Small sized vessels (30%)
Biopsy	Often negative	Usually positive
		Granulomatous: (80%)
		Lymphocytic: (20%)
		Evidence of amyloid-beta: > 50%
CSF	Pleocytosis in 35%	Pleocytosis in approximately 60–65%
MRI/lesion pattern	Possibly downstream territorial infarcts	Multiple; especially white matter lesions (WML), small (lacunar) infarcts
DSA/MRA	Usually pathological findings: abnormal/irregular vascular lumina; vessel wall enhancement on T1w after CM administration	≤ 30% pathological findings: abnormal/irregular vascular lumina

Abbreviations: *CM* Contrast medium, *CSF* Cerebrospinal fluid, *DSA* Digital subtraction angiography, *MRA* Magnetic resonance angiography, *MRI* Magnetic resonance imaging

### ANCA (Antineutrophilic Cytoplasmatic Antibodies) Associated Vasculitides

Aetiology: The most important representatives are granulomatosis with polyangiitis (GPA; formerly Wegener’s granulomatosis) and eosinophilic granulomatosis with polyangiitis (EGPA; formerly Churg-Strauss syndrome) (Table 1; [1, 11, 59–65]). Autoantibodies target endogenous leukocyte antigens and especially in GPA, autoantibodies are directed against proteinase 3 (PR3-ANCA) [1, 3, 11]. Moreover, additional environmental and genetic factors are not yet fully understood.

**Fig. 8:**
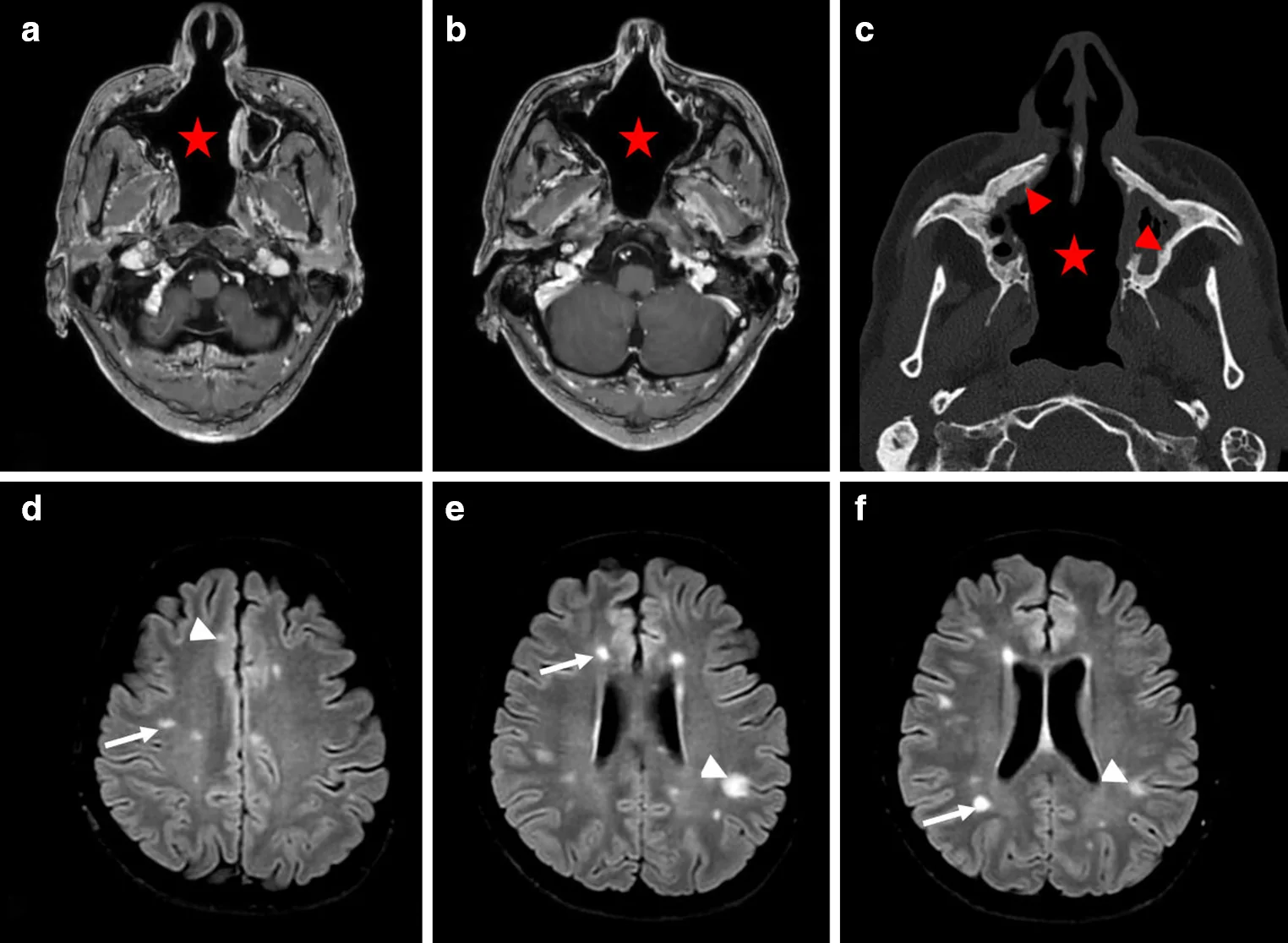
Granulomatosis with polyangiitis (GPA; Wegener’s granulomatosis). **a**–**c** Extended destruction of the paranasal sinuses and the nasal septum (**a**, **b** post contrast T1 WI; **c** CT; asterisk) with increased sclerosis of adjacent bony structures (**c** arrowhead); **d**–**f** fluid attenuated inversion recovery (FLAIR) images showing numerous subcortical (**d**–**f** arrow) and also juxta-/cortical lesions (**d**–**f** arrowhead)

Key clinical features: GPA is a necrotizing vasculitis affecting skin and mucosa and is associated with severe destructive lesions of the oral, nasal, and pharyngeal regions (Fig. 8; [1, 11, 59, 60]). Imaging pattern: CNS involvement occurs in approximately 50% of cases and manifests as nonspecific T2-hyperintense lesions, meningeal and parenchymal granulomas, and infarctions (Fig. 8; [59, 60]).

**Fig. 9:**
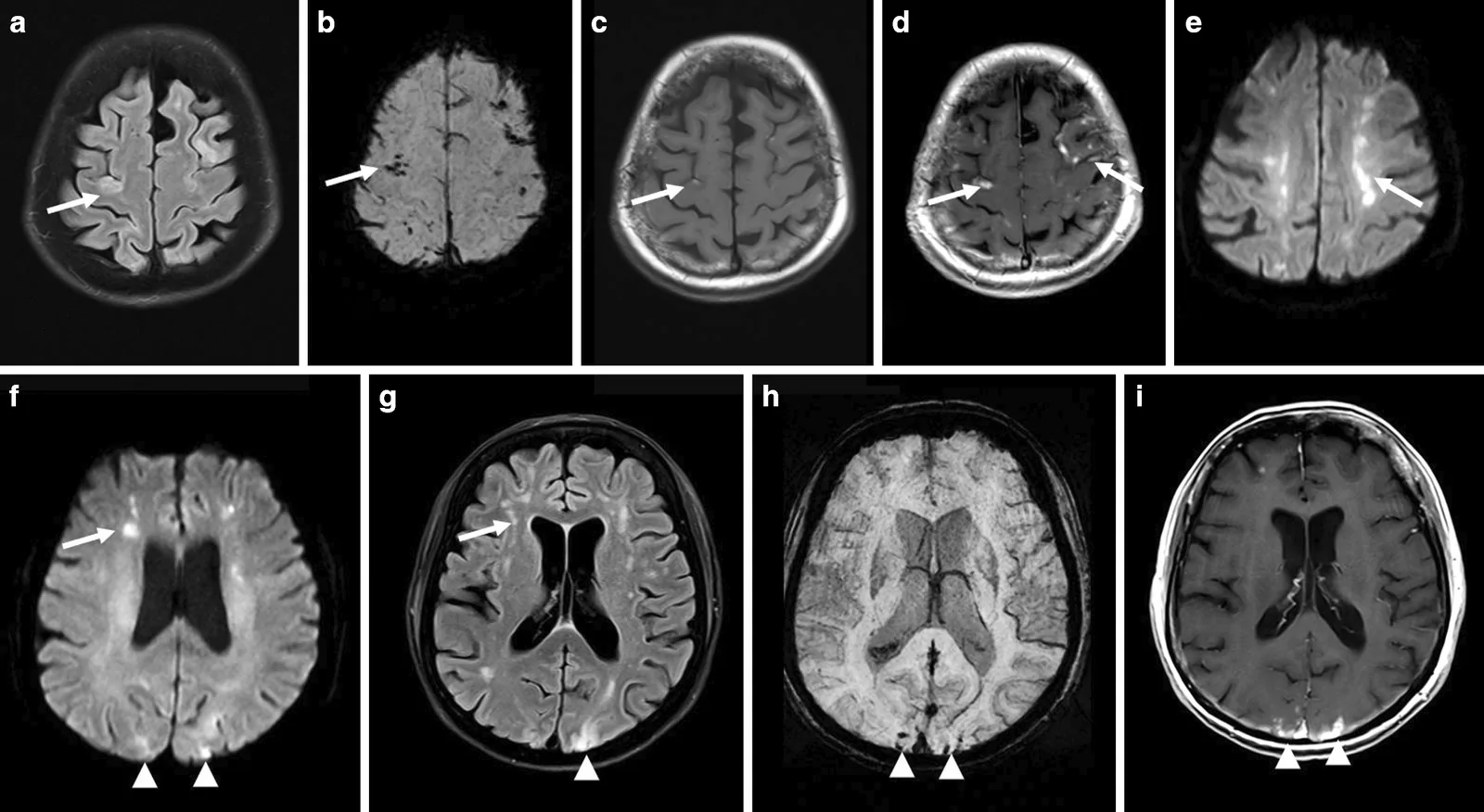
Eosinophilic granulomatosis with polyangiitis (EGPA; Churg-Strauss syndrome) in a 65-year old woman suffering from progressive amnestic syndrome and slight right-sided hemiparesis. **a**–**e** bilateral cortical and subcortical hyperintense lesions (**a**, **g** arrow; fluid attenuated inversion recovery (FLAIR) images) with punctuate signal loss (**b** arrow; susceptibility weighted imaging (SWI)) and hyperintense signal on T1 WI (**c** arrow), inhomogeneous enhancement on post contrast (pc) T1 WI (**d** arrow); bilateral diffusion restriction on (**e**, **f** arrow; DWI, b = 1000 s/mm^2^); **f**–**i** additional occipital sulcal conglomerations (**f**–**i** arrowhead) with distinct contrast enhancement (**i** , arrowhead), signal loss on SWI (**h** arrowhead), diffusion restriction (**f** arrowhead) and hyperintense signal on FLAIR images (**g** arrowhead)

EGPA is characterized by eosinophilic granulomas in medium-sized and small vessels and necrotizing vasculitis. Key clinical features: A hallmark symptom is allergic asthma due to pulmonary involvement [11, 62, 63]. Other organs commonly affected include skin, kidneys, paranasal sinuses, and the nervous system. The peripheral nervous system is involved in up to 70% of cases, typically presenting as mononeuritis multiplex [11, 62, 63]. Imaging pattern: CNS involvement may result in infarctions, haemorrhages, meningeal alterations, and cranial nerve involvement (Fig. 9; [9, 11]). Laboratory findings include increased levels of Ig (Immunoglobulin) E, complement C3 and C4, as well as eosinophilia, which may also be present in the CSF [1, 9, 11, 62–65]. The most important differential diagnoses are listed in Fig. 1.

- Ig (Immunoglobulin) G 4 related diseases (RD)

**Fig. 10:**
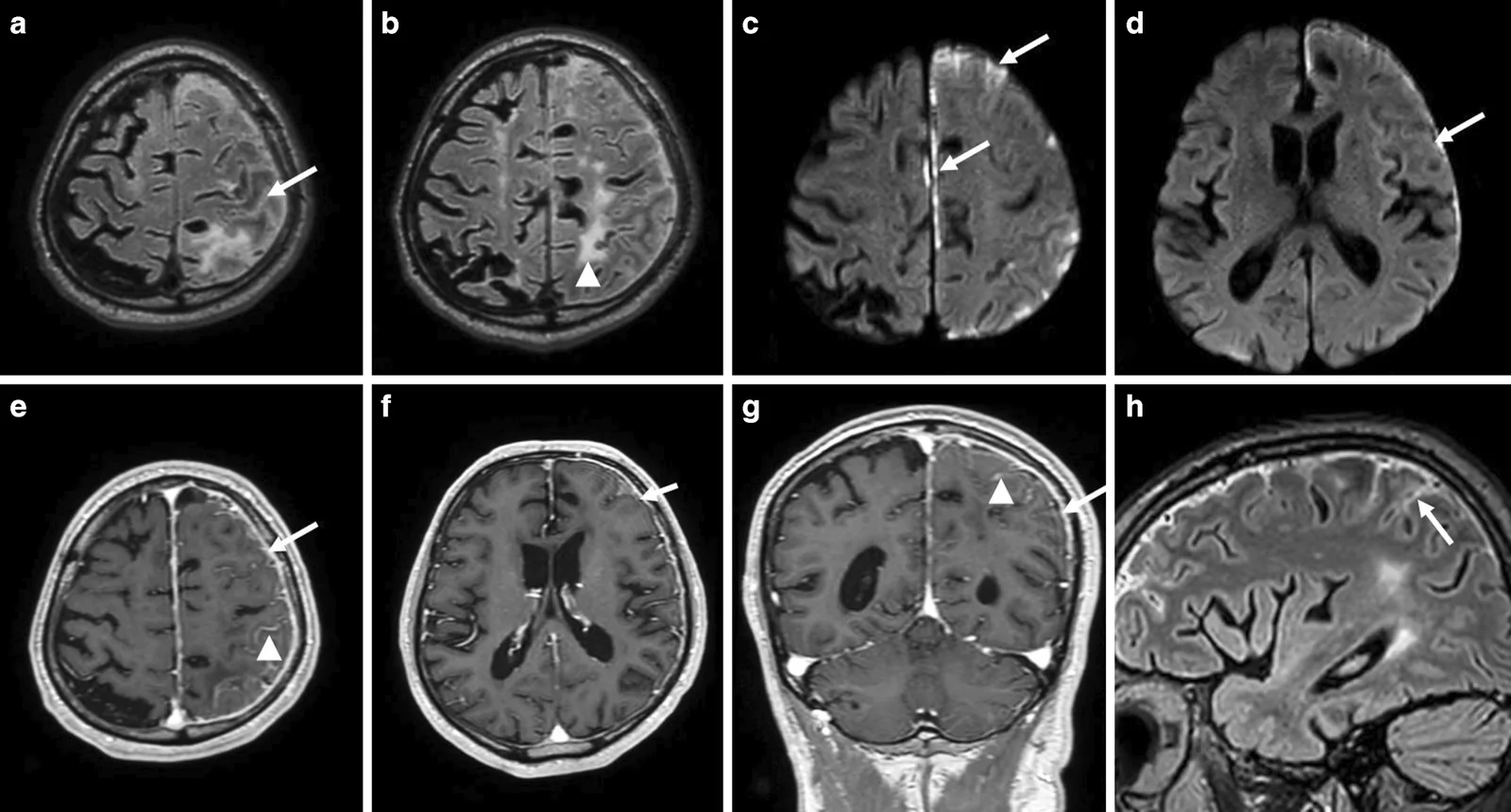
IgG 4 related biopsy proven hypertrophic pachymeningitis in a 61 year old man with slight right-sided hemiparesis and first epileptic seizure. **a**–**h** sulcal effusion (**a**, **h**): arrow; fluid attenuated inversion recovery (FLAIR) and adjacent cortical and subcortical hyperintense signal changes (**b** arrowhead); diffusion restriction (**c**, **d** arrow; diffusion weighted imaging (DWI), b = 1000 s/mm^2^) and distinct contrast enhancement (CE) of the thickened pachymeninx (**e**–**g** arrow). Note also slight CE of leptomeningeal structures (**e**, **g** arrowhead)

**Fig. 11:**
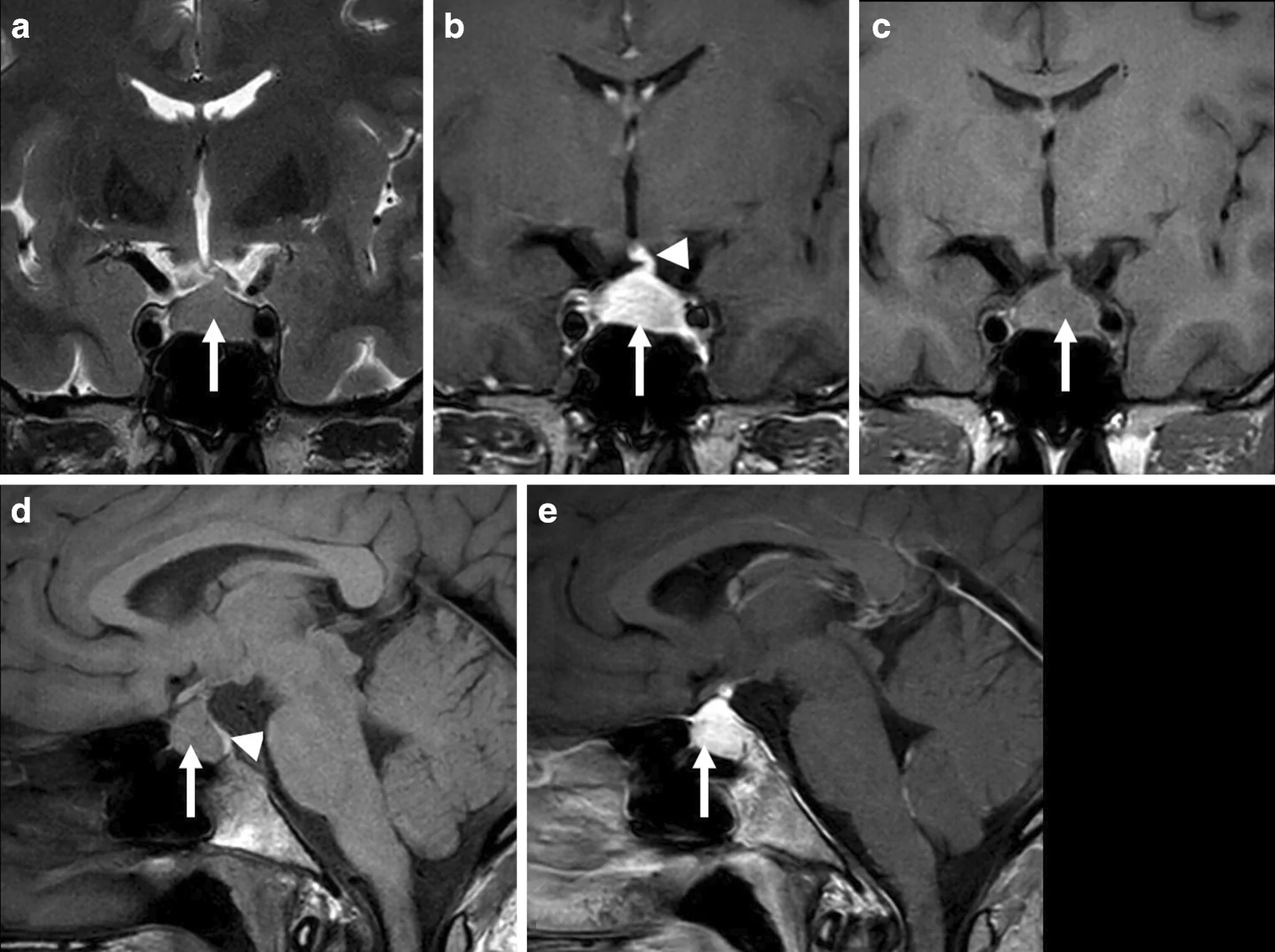
IgG 4 related biopsy proven hypophysitis. **a**–**e** Enlarged pituitary gland (**a**–**e** arrow), thickened pituitary stalk (**b** arrowhead) and homogeneous contrast enhancement (**b**, **e**); note hyperintense signal of the neurohypophysis (**d** arrowhead) on sagittal T1 weighted images (WI)

**Fig. 12:**
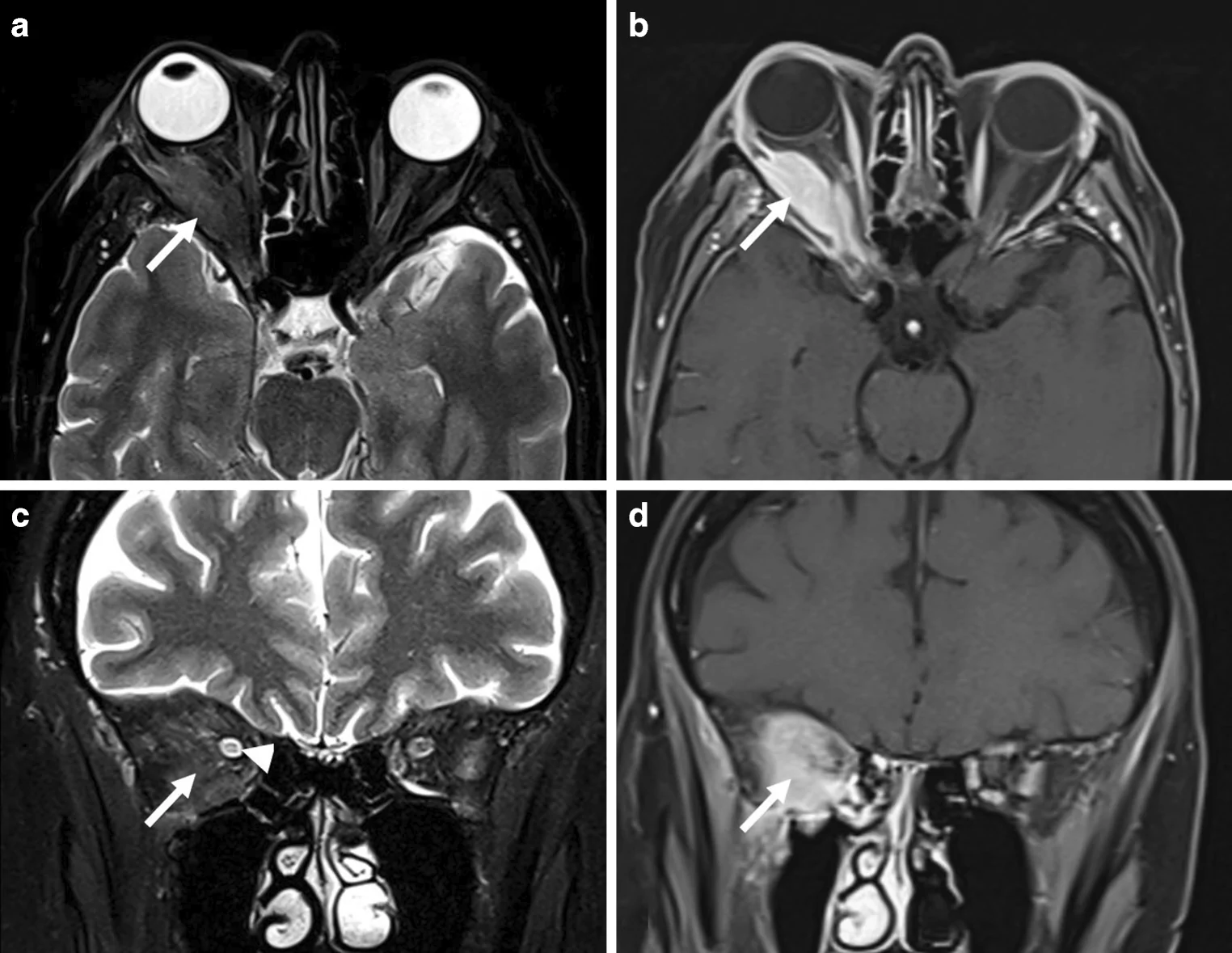
IgG 4 related orbital pseudotumor in a 55 year old woman suffering from progressive right-sided visual impairment and double vision. **a**–**d** axial (**a**) and coronal (**c**) T2 weighted images (WI) showing an inhomogeneous hypointense right-sided orbital mass lesion (**a**, **c** arrow), exophthalmos and optic nerve atrophy (**c** arrowhead); **b**, **d** post contrast T1 WI disclose intense contrast enhancement (arrow)

Aetiology: Although the pathophysiology of IgG 4‑RD is up to now not fully understood, the focus of this disease is particularly on the interaction of five immune cell populations, namely B lymphocytes and plasma cells, CD4 T lymphocytes and T helper cells, and M2 macrophages [33–35, 66]. This results in chronic inflammation, which stimulates fibroblasts to produce collagen-rich extracellular matrix. In consequence, IgG 4‑RD show the characteristic signal reduction in T2-weighted sequences [33–35, 67–74]. The course is usually slowly progressive, and fulminant variants are rare. There is no specific serum marker in laboratory tests. In approximately 50% of cases, IgG 4 is negative, meaning that a biopsy is often required to determine the aetiology. Moreover, in approximately 50% of cases, phlebitis is observed on biopsy, meaning that IgG 4‑RD are currently classified as vasculitides [33–35, 66]. However, IgG 4 can also be positive in other granulomatous diseases with infectious aetiology, in sarcoidosis or even in malignancies. So-called ‘overlap syndromes’ occur particularly in ANCA positive vasculitides [34, 35]. In general, enlarged organs are observed, with a distinction being made between a pancreatic-hepatic-biliary type, a retroperitoneal type and head and neck type [33–35]. Imaging pattern: In the latter type, the pachymeninges (Fig. 10), the pituitary gland and the pituitary stalk (Fig. 11), the cavernous sinus and in addition the lacrimal gland as well as intraorbital structures (Fig. 12) may be affected.

*Hypertrophic pachymeningitis: *Histopathological examination reveals lymphoplasmacytic infiltrates, particularly in the pachymeninges [33–35, 68]. MRI shows thickening of the dura mater with marked enhancement in T1 WI after contrast agent administration (Fig. 10; [69–73]). However, the differential diagnosis is broad and includes inflammatory, neoplastic, metabolic-toxic and mechanical conditions such as spontaneous intracranial hypotension (SIH), sequelae of neurosurgical procedures, subdural haematomas and hygromas, and venous drainage disorders [71, 72]. While slight enhancement of the dura mater over short segments, the falx and the tentorium occurs physiologically, enhancement of the leptomeninges is always pathological [71, 72].

b) Idiopathic orbital inflammation (IOI)

IOI also known as “nonspecific orbital inflammation” or “orbital inflammatory pseudotumor” is a diagnosis of exclusion, not due to any known etiology or systemic illness [75, 76]. Therefore, in all patients with suspected IOI complete ophthalmic assessment is crucial [75–77]. Tolosa-Hunt syndrome (THS) has been considered a subtype of IOI [75, 76].

### Tolosa-Hunt Syndrome (THS)

**Fig. 13:**
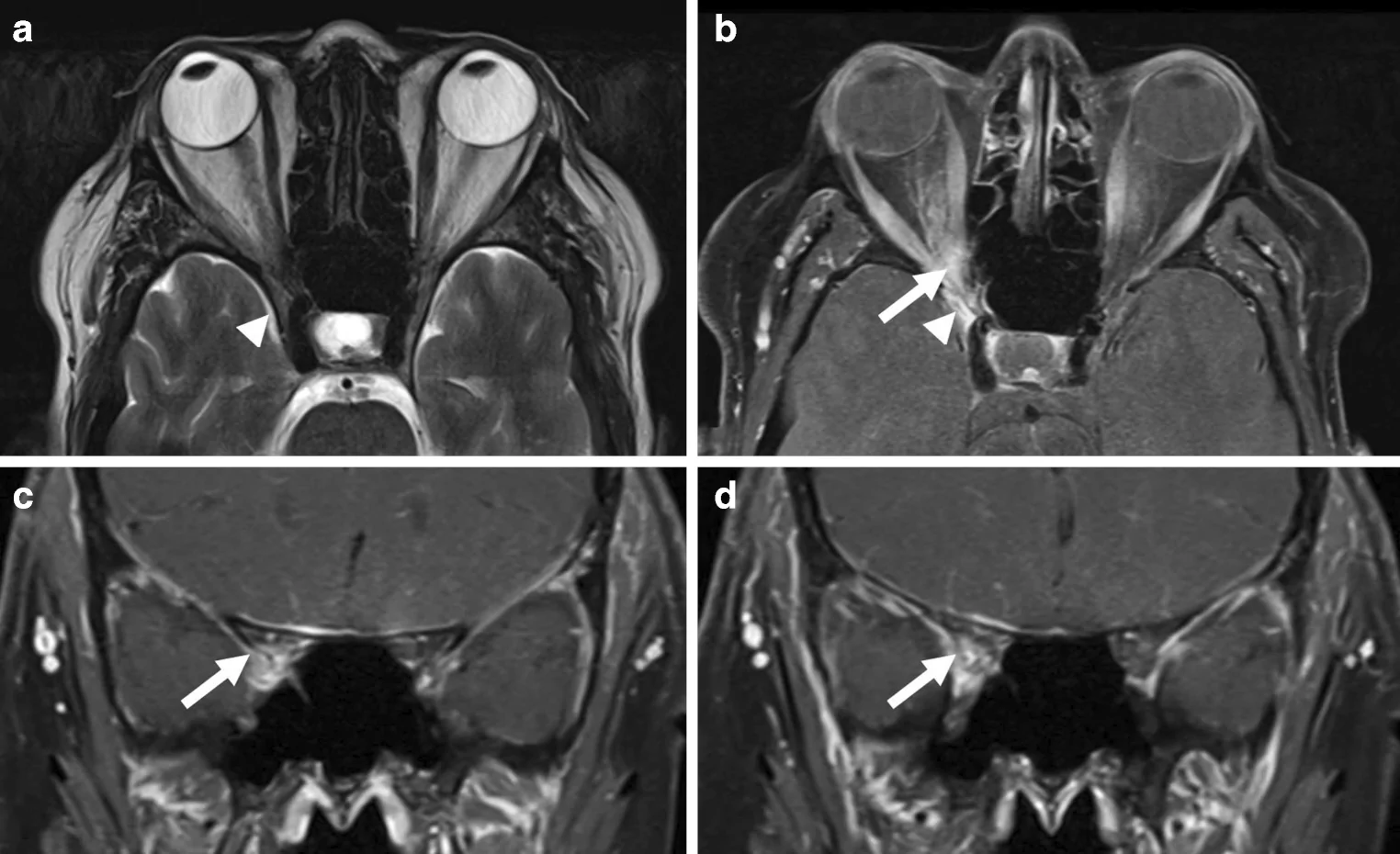
Tolosa-Hunt syndrome. **a** Circumscribed hyperintense lesion in the right lateral cavernous sinus (**a** arrowhead; T2 weighted images (WI)); **b**–**d** partially inhomogeneous contrast enhancing (CE) lesion in the right anterolateral cavernous sinus (**b** arrowhead) as well as in the orbital apex (**b**–**d** arrow)

Aetiology: THS is caused by granulomatous lymphocytic inflammation in the cavernous sinus, the superior orbital fissure and the orbital apex [36, 37]. Key clinical features: Neurologically, subacute peri- and intraorbital pain occurs, accompanied by paresis of cranial nerves III, IV and VI and in addition sensory disturbances in the area innervated of the first trigeminal branch (ophthalmic nerve) (Fig. 13; [36, 37, 78-80]). The second trigeminal branch (maxillary nerve) and the optic nerve (CN II) may also be involved, but less frequently. Besides neurological disturbances and imaging features an important diagnostic criterion of the International Headache Society (IHS) is rapid remission of pain within 72 h after initiation of glucocorticoid therapy [78, 79]. This drug response can occasionally be used to distinguish IgG 4 related cavernous or orbital involvement, which, however, also may respond well to glucocorticoid administration in the early stages before increased fibrosis (Fig. 12; [81–83]).

Imaging pattern: Primary criteria in MRI are a) a lesion in the anterior cavernous sinus, b) an increase in the size of the cavernous sinus and c) lateral wall protrusion. Secondary criteria are a) involvement of the internal carotid artery and/or the orbital apex and the optic nerve, b) extension into the superior orbital fissure, and c) a lack of sinus wall demarcation in T2 WI (Fig. 13; [80, 84]). The differential diagnoses of painful most often incomplete ophthalmoplegia are listed in Table 5; [79]. Accordingly, other causes must be ruled out before a diagnosis of THS can be made (see Fig. 1).

**Table 5:** Differential diagnosis of painful ophthalmoplegia

Etiology	Cause
a) vascular	ICA aneurysm at the origin of the posterior communicating artery (ophthalmoplegic aneurysm/CN III)
	ICA aneurysm in the cavernous segment (CN VI)
	Carotid-cavernous fistula (direct, indirect)
b) diabetogenic	Especially CN III > CN VI
c) inflammatory/infectious	Fungi, tuberculosis, herpes zoster, other (differential diagnosis: orbital phlegmone)
d) inflammatory/autoimmune	Poly-(Oligo-)neuritis cranialis
	Sarcoidosis
	ANCA—associated vasculitis (AAV: GPA, EGPA)
	Giant cell arteritis (arteritis cranialis)
	IgG 4—related diseases
	Idiopathic orbital inflammation (IOI), e.g., Tolosa-Hunt syndrome
	other
e) ophthalmoplegic migraine	–
f) neoplastic lesion	Tumors of the skull base, lymphoma, metastasis, other

Abbreviations: *CN* Cranial nerve, *EGPA* Eosinophil granulomatosis with polyangiitis, *GPA* Granulomatosis with Polyangiitis, *ICA* Internal carotid artery

## Infectious Diseases

The spectrum of infectious granulomatous CNS diseases is broad and includes bacterial, parasitic, and fungal infections (Table 1 and Fig. 1; [2]). For clarity, the most important bacterial and parasitic representatives, i.e., neurotuberculosis [85–91], neurosyphilis [92–95] and CNS toxoplasmosis [96–98] are discussed below, with reference to specific literature for further details [87, 96, 99].

- Neurotuberculosis

Aetiology: Due to increased migration and globalization, the prevalence of neurotuberculosis in Western European countries is rising again. However, diagnosis may be challenging because of initially nonspecific and diverse manifestations. CNS involvement occurs in approximately 2–5% of patients with tuberculosis [85, 86, 88]. The initial lesions are small caseating tubercles (“Rich foci”), resulting from haematogenous dissemination and located anywhere in the CNS or meninges [2, 3, 85, 86]. The T‑cell mediated granulomatous inflammatory response limits mycobacterial spread, forming epithelioid cells, Langhans giant cells, and sometimes central caseation within the granulomas [85, 86, 90]. Tuberculous meningitis caused by rupture of granulomas with subsequent bacillary dissemination is the most common and most severe manifestation of neurotuberculosis [88, 89].

**Fig. 14:**
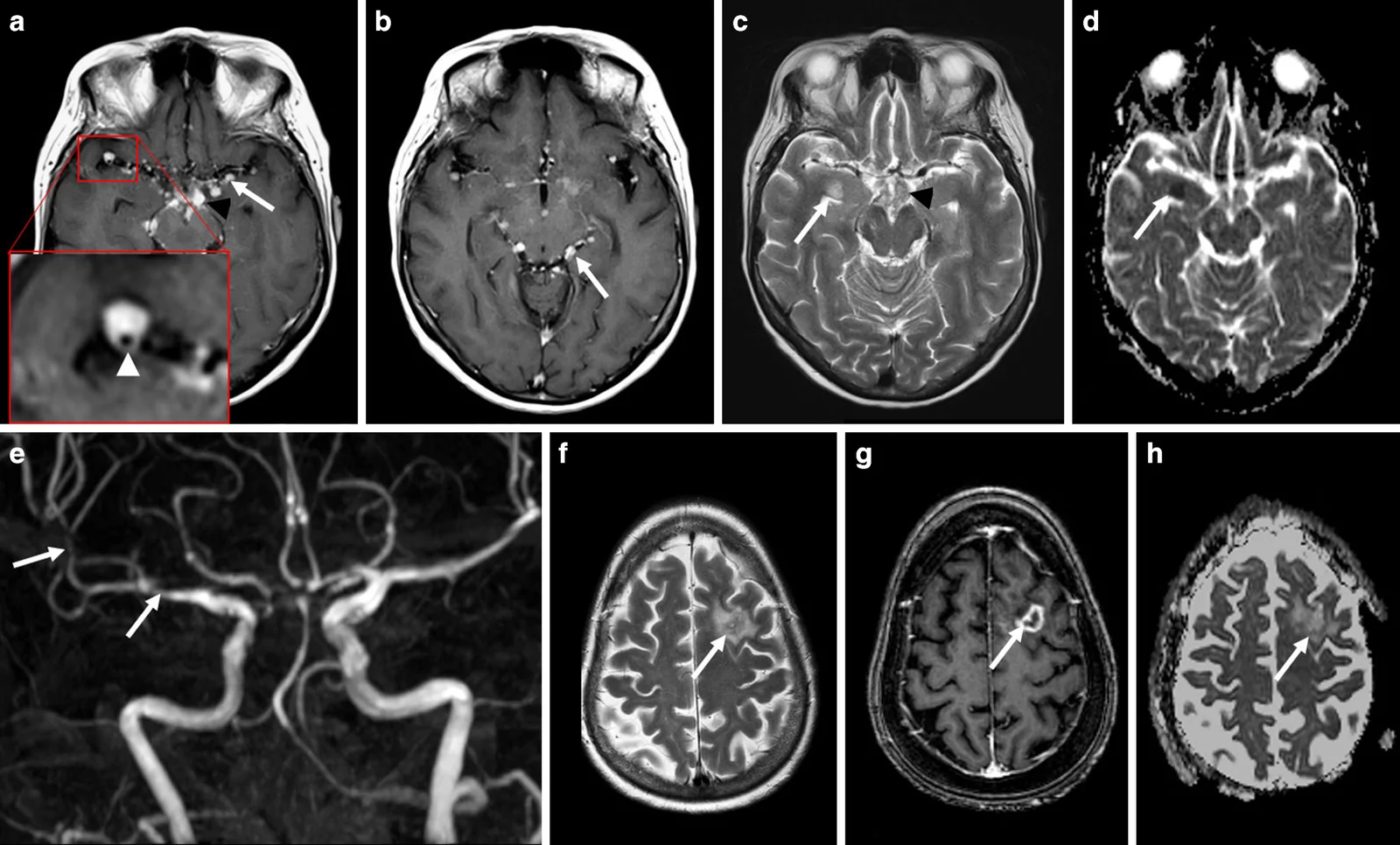
CNS tuberculosis. **a**–**e** multiple nodular enhancing lesions especially in the basal cisterns (**a**, **b** arrow; post contrast T1 weighted images (WI)), partially encasing basal arteries (**a** box: arrowhead) and cranial nerves (**a**, **c** arrowhead; oculomotor nerve); small temporal infarct (**c**, **d** arrow; **c** T2 WI; **d** DWI/apparent diffusion coefficient (ADC) map; ADC-value: 0.523 ± 0.038 × 10^−3^ mm^2^/s; mean ± standard deviation (SD)) and several vascular stenoses on MR angiography (**e** arrow); **f**–**h** intraaxial tuberculous granuloma (arrow) frontodorsal left with inhomogeneous central T2 hyperintense (**f**) und T1 hypointense (**g**) signal changes, marginal contrast enhancement (**g** arrow) and partially increased ADC-value (1.44 ± 0.12 × 10^−3^ mm^2^/s; mean ± SD; **h** arrow) indicating caseating tuberculoma with liquefaction

Imaging pattern: Due to the predominance of basal exudates (basal meningitis), vessels and cranial nerves in the basal subarachnoid space are preferentially affected [85, 86, 88]. Cranial nerve (CN) palsies particularly of CN III and VI occur in 25–50% of cases [85, 88]. Whereas vasospasm may mediate ischemic lesions in early stages of the disease, in later stages a localized tuberculous vasculitis with transmural inflammation and consecutive reduction of the lumina is discussed (Fig. 14; [85, 86, 89, 91]). Most often the vascular territories of the basal perforators originating from the M1-segment of the middle cerebral artery (MCA) and from the circle of Willis are affected [86, 88, 89]. This may lead to infarctions, especially in the basal ganglia and thalami (Fig. 14). Non-communicating hydrocephalus occurs in approximately 66% due to basal meningeal adhesions [86, 88]. Further manifestations of neurotuberculosis include dural adherent or intraparenchymal tuberculoma, less commonly tuberculous abscess, and also extra- or intradural spinal involvement [90]. The most important differential diagnoses, particularly taking into account overlapping imaging findings, are listed in Fig. 1.

b) Neurosyphilis

**Fig. 15:**
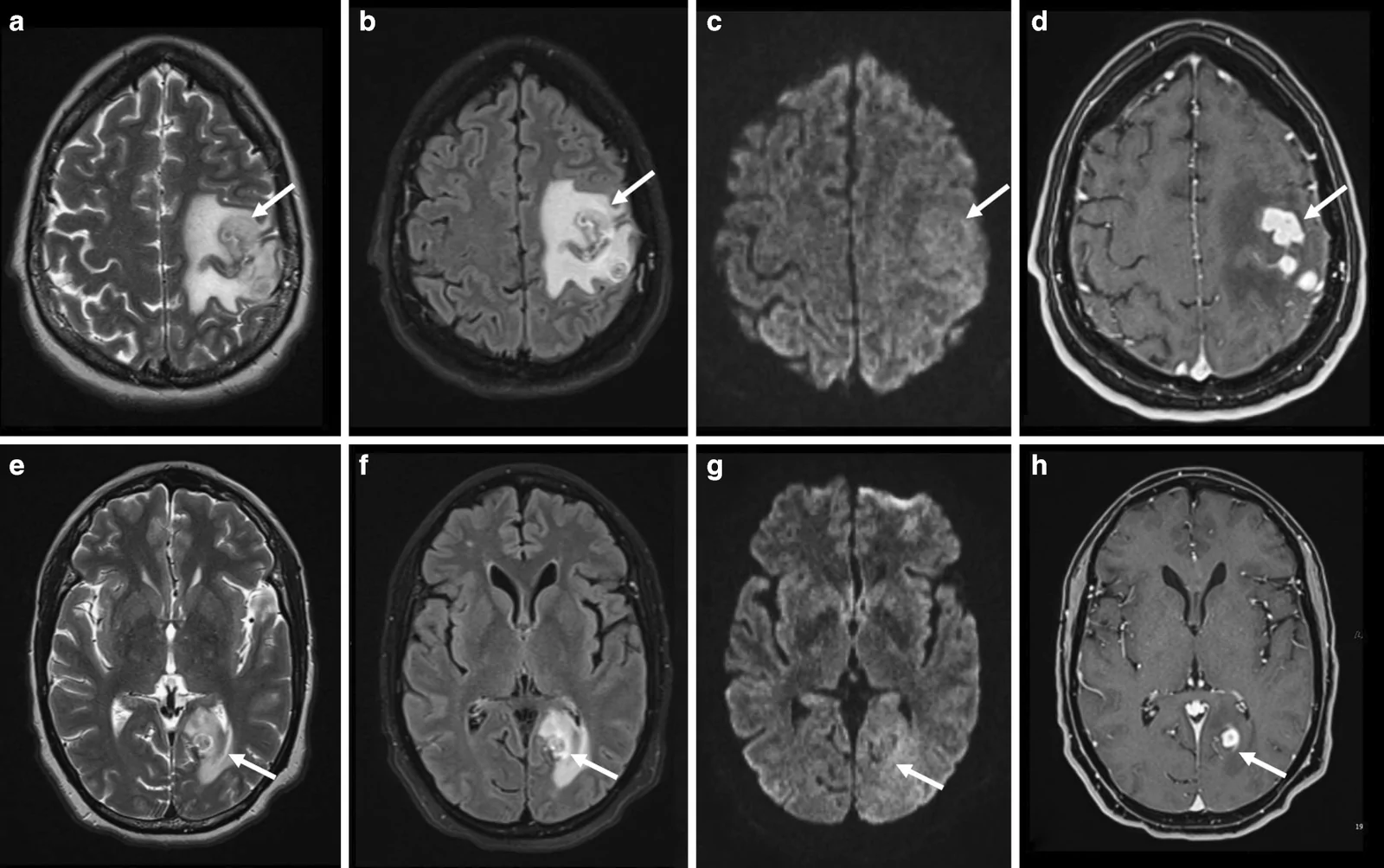
Neurosyphilis. A 50-year old man positive for HIV suffering from progressive right-sided hemiparesis and recurrent epileptic seizures. **a**–**h** MRI showing several lesions in the pre- and postcentral region (**a**, **b** arrow) without restricted diffusion (**c** arrow; diffusion weighted imaging (DWI); b = 1000 s/mm^2^) and distinct contrast enhancement on T1 WI (**d** arrow); **e**–**h** similar lesion left-sided paramedian occipital (**e**–**h** arrow). Histological examination proved syphilitic gummata

Key clinical features: Clinically, three stages are distinguished: primary/secondary, latent, and tertiary (late) syphilis [92]. In the tertiary stage three CNS manifestations are recognized additionally: a) meningovascular type, associated with cerebral infarctions, polyradiculitis, and granulomas (gummata) (Fig. 15), b) progressive paralysis with progressive neuropsychiatric symptoms, and c) tabes dorsalis, predominantly affecting the posterior columns of the spinal cord [87, 92, 93, 95].

**Fig. 16:**
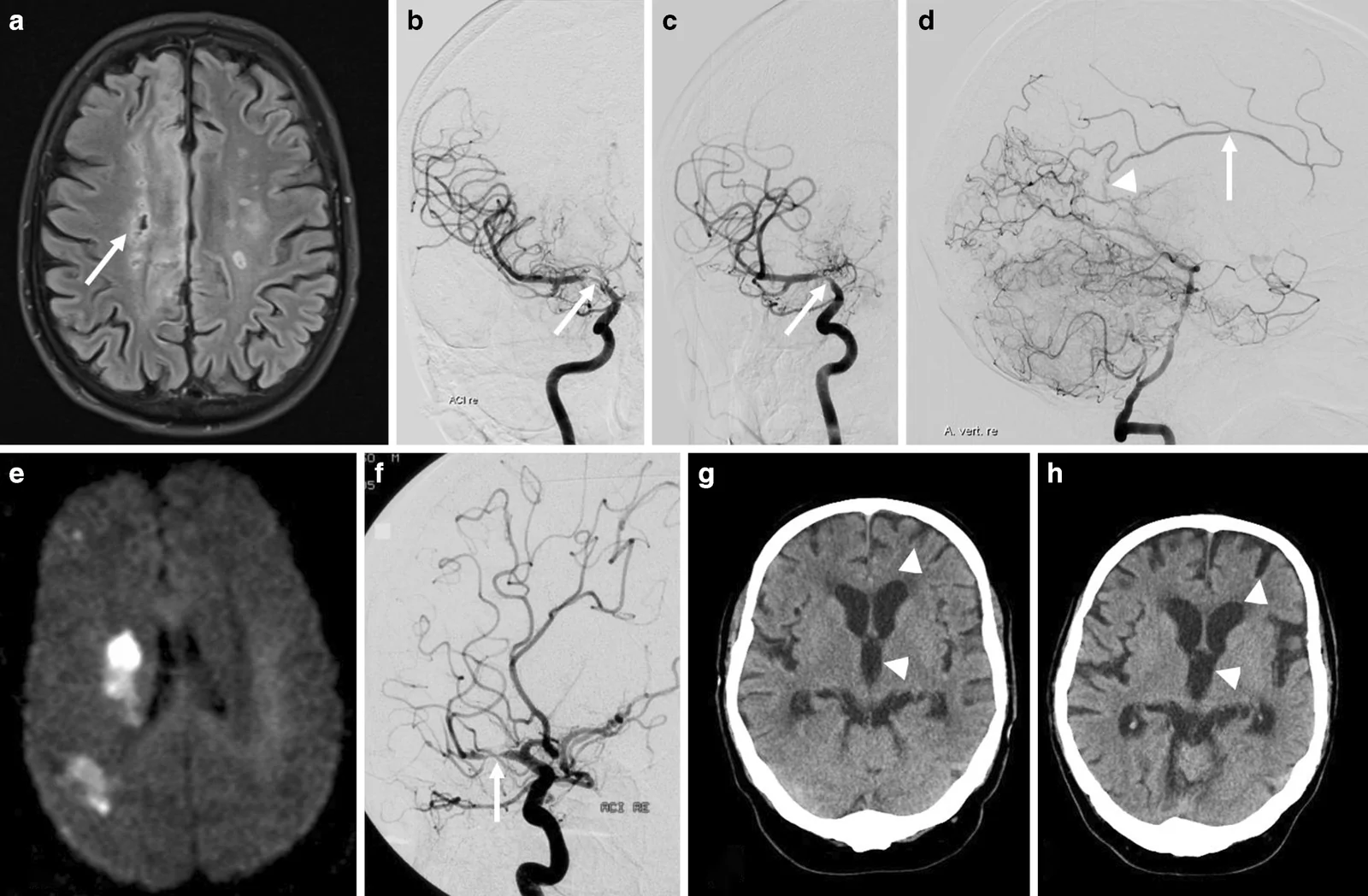
Neurosyphilis. **a**–**f** meningovascular type: hemodynamic (**a** arrow) and territorial infarcts (**a** arrowhead; fluid attenuated inversion recovery (FLAIR) images) due to severe distal internal carotid artery (ICA; C7-segment) stenosis and the proximal right-sided A1- and M1-segment (**b**, **c** arrow; Heubner arteritis); retrograde filling of the pericallosal artery (**d** arrow) via leptomeningeal collaterals as well as via posterior artery branches of the corpus callosum (**d** arrowhead); **e**, **f** acute infarcts (**e**; diffusion weighted imaging (DWI); b = 1000 s/mm^2^) due to vasculitic right-sided M1 stenosis (**f** arrow); **g**, **h** progressive paralysis of neurosyphilis resulting in rapid progressive brain atrophy within 9 months in a 61-year old woman (arrowheads)

Imaging pattern: MRI may show nodular thickening of the meninges with increased post-contrast T1 enhancement (Fig. 1; [92]). Predilection sites include the brainstem, cerebellum, skull base regions, and cranial nerves, particularly the optic nerve leading to secondary atrophy [92–95]. In addition, diffuse or frontotemporal-predominant cerebral atrophy (progressive paralysis) and infarctions due to pathogen-associated vasculitis (Heubner arteritis) [2, 3, 87, 92], typically in the middle cerebral artery territory, may be observed (Fig. 16).

c) CNS toxoplasmosis

**Fig. 17:**
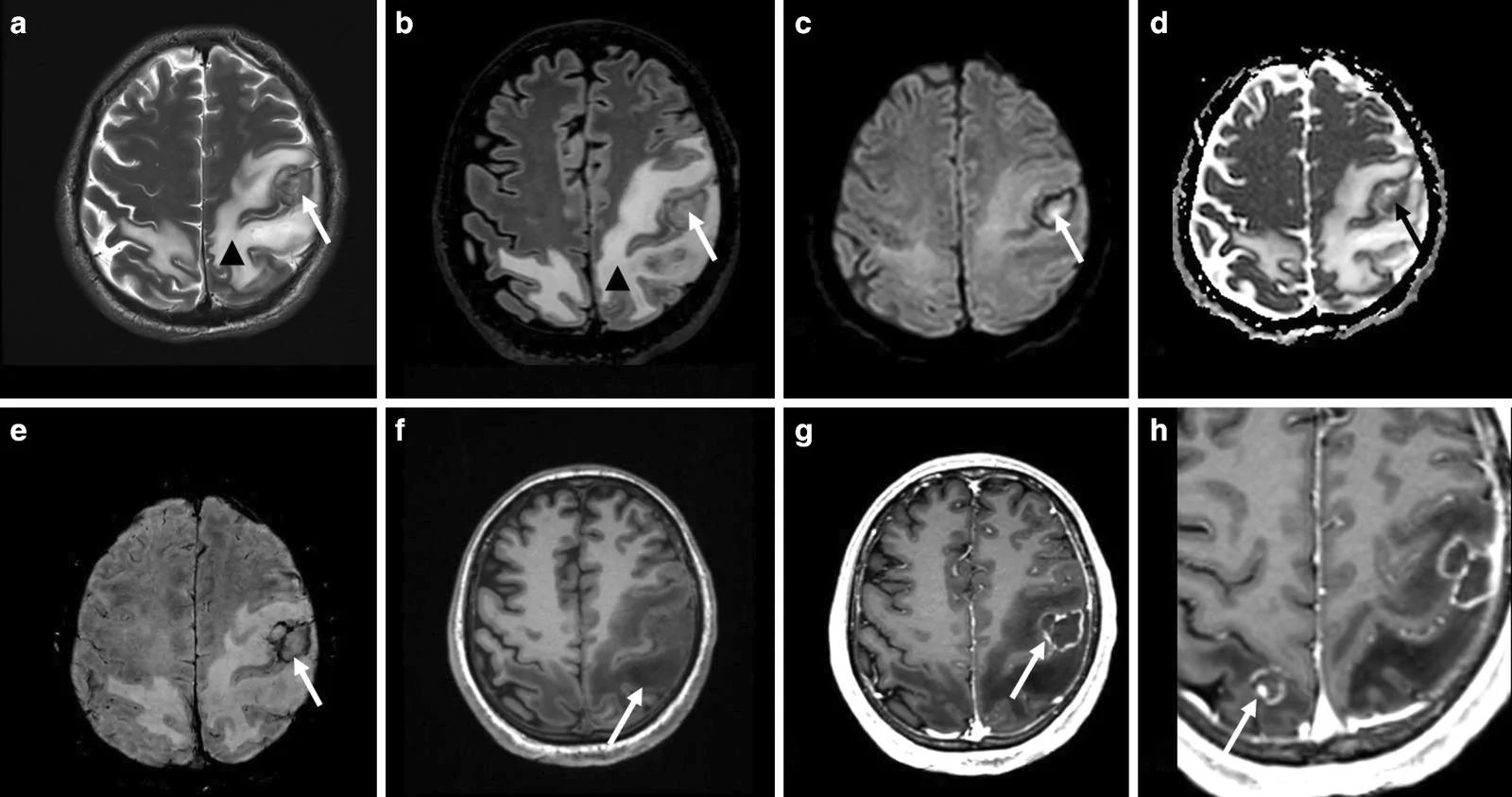
Toxoplasmosis. 39-year old man with HIV infection suffering from right-sided progressive hemiparesis and recurrent epileptic seizures. **a**–**g** inhomogeneous hyperintense lesion in the left-sided central region on T2 weighted images (WI) (**a**, **b** arrow; **b** fluid attenuated inversion recovery (FLAIR)) with extensive perifocal edema (**a**, **b** black arrowhead), restricted diffusion (**c**, **d** arrow; **c** DWI; **d** apparent diffusion coefficient (ADC) map; ADC-value: 0.703 ± 0.042 × 10^−3^ mm^2^/s; mean ± SD; **d** arrow); marginal signal loss on susceptibility WI (SWI) (**e** arrow) and inhomogeneous contrast enhancement (**f**, **g** arrow; **f** T1 WI; **g**, **h** post contrast T1 WI); note excentric target sign in another right-sided postcentral/parietal lesion (**h** arrow)

**Fig. 18:**
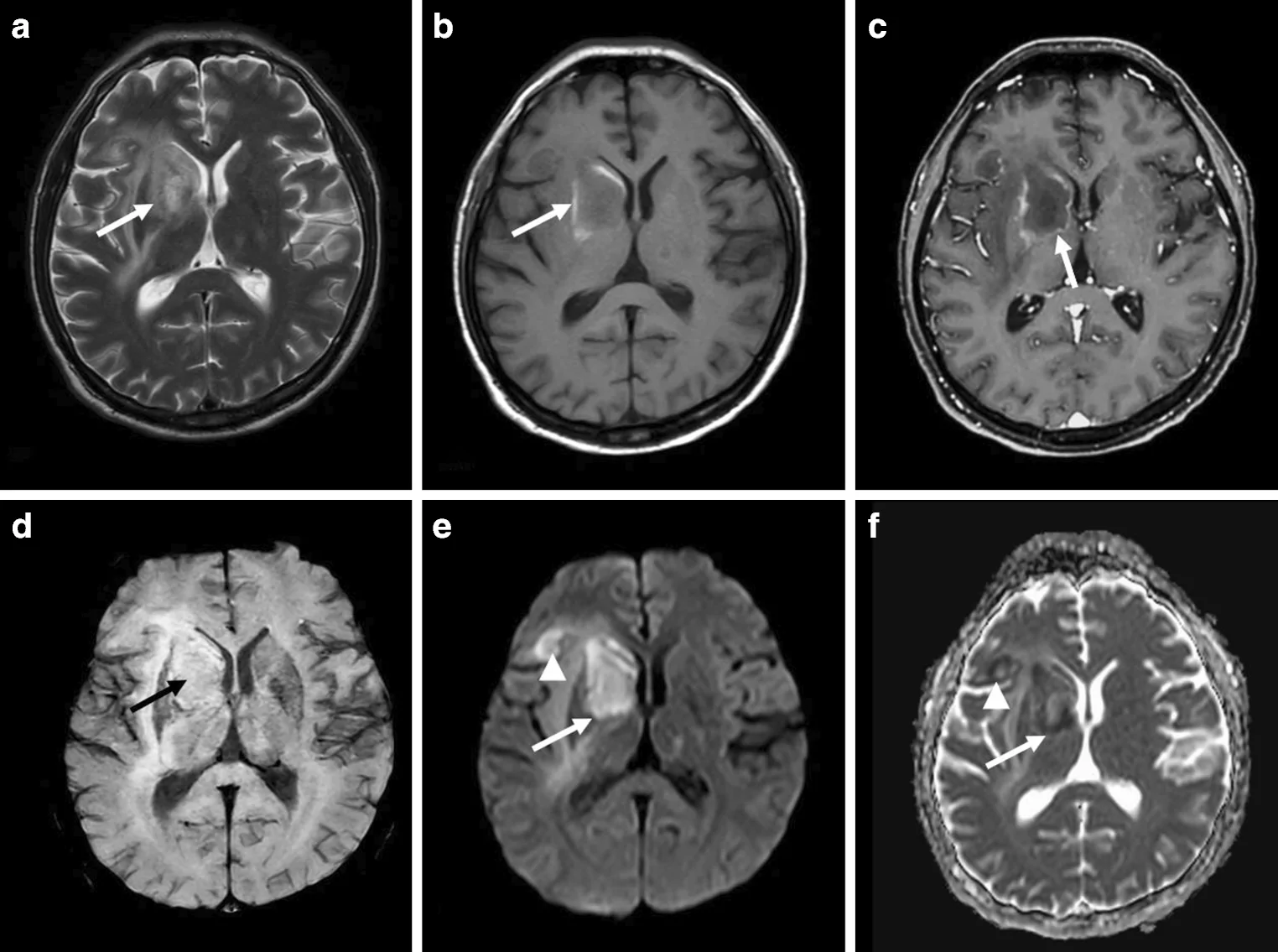
Toxoplasmosis (continued). **a**–**f** Large lesion in the right basal ganglia exhibiting concentric target sign on T2 weighted images (WI; **a** arrow); incomplete hyperintense rim on T1 WI (**b** arrow), slight enhancement (**c** arrow), no signal loss on SWI (**d** arrow) and marginal restricted diffusion (**e**, **f** arrow, arrowhead; **e**, **f** DWI, b = 1000 s/mm^2^, apparent diffusion coefficient (ADC) map)

Aetiology: The endemic infection rate increases up to 70–80% (65 years of age) with age due to uptake of oocytes by contact with cat excrement or through tissue cysts (bradyzoites) during consumption or handling of raw meat [96, 98]. Whereas the pathogens normally cannot cause CNS infection in immunocompetent individuals, CNS toxoplasmosis is a typical infectious manifestation in immunodeficient patients (Fig. 1; [87, 96-98]). Imaging pattern: The often multiple CNS lesions are located predominantly in the cortical or deep grey matter. MRI discloses pronounced perifocal edema [98]. Beside concentric target sign on T2 WI (Figs. 17 and 18), T1 WI after contrast medium application may exhibit eccentric target sign in up to 30% (Fig. 18; [100, 101]). From a histopathological point of view the central enhancing core of eccentric target sign seems to represent inflamed leaky vessels in the sulcus entering into the lesion [100]. Furthermore, unlike bacterial abscesses, toxoplasmosis lesions often show only moderate diffusion restriction [100].

## Proliferative Neoplastic Disorders

- Langerhans Cell Histiocytosis (LCH)

**Fig. 19:**
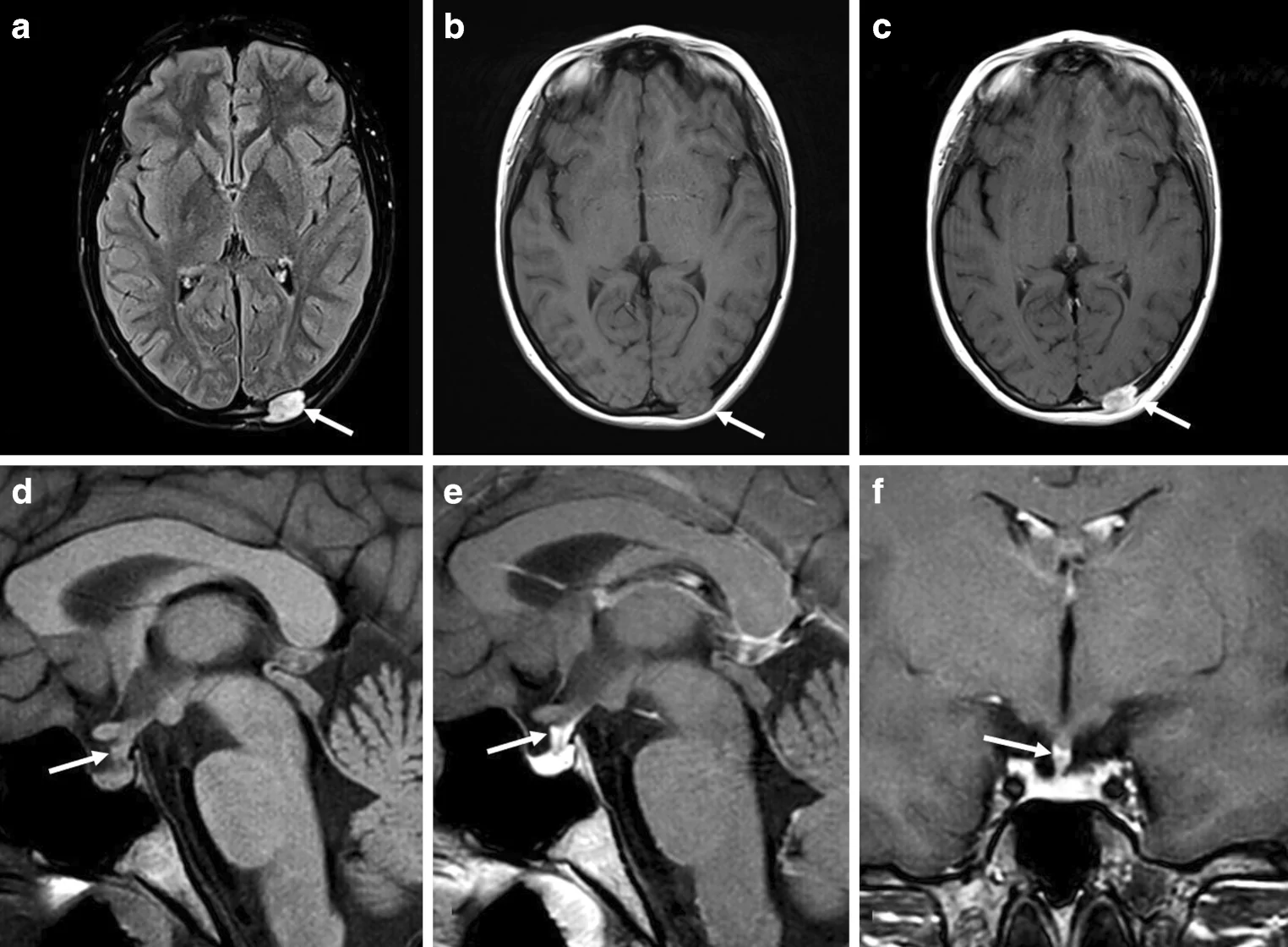
Langerhans cell histiocytosis (LCH). **a**–**c** 13 year old boy suffering from progredient occipital tumescence. Left-sided hyperintense intraosseous occipital lesion (**a** arrow; fluid attenuated inversion recovery (FLAIR) images) with distinct contrast enhancement (CE) (**b**, **c** arrow; T1 weighted images (WI) pre- and post-contrast); **d**–**f** 15-year old boy with growth arrest since two years. Thickened pituitary stalk (**d**–**f** arrow) with intense CE (**e**, **f** arrow)

**Fig. 20:**
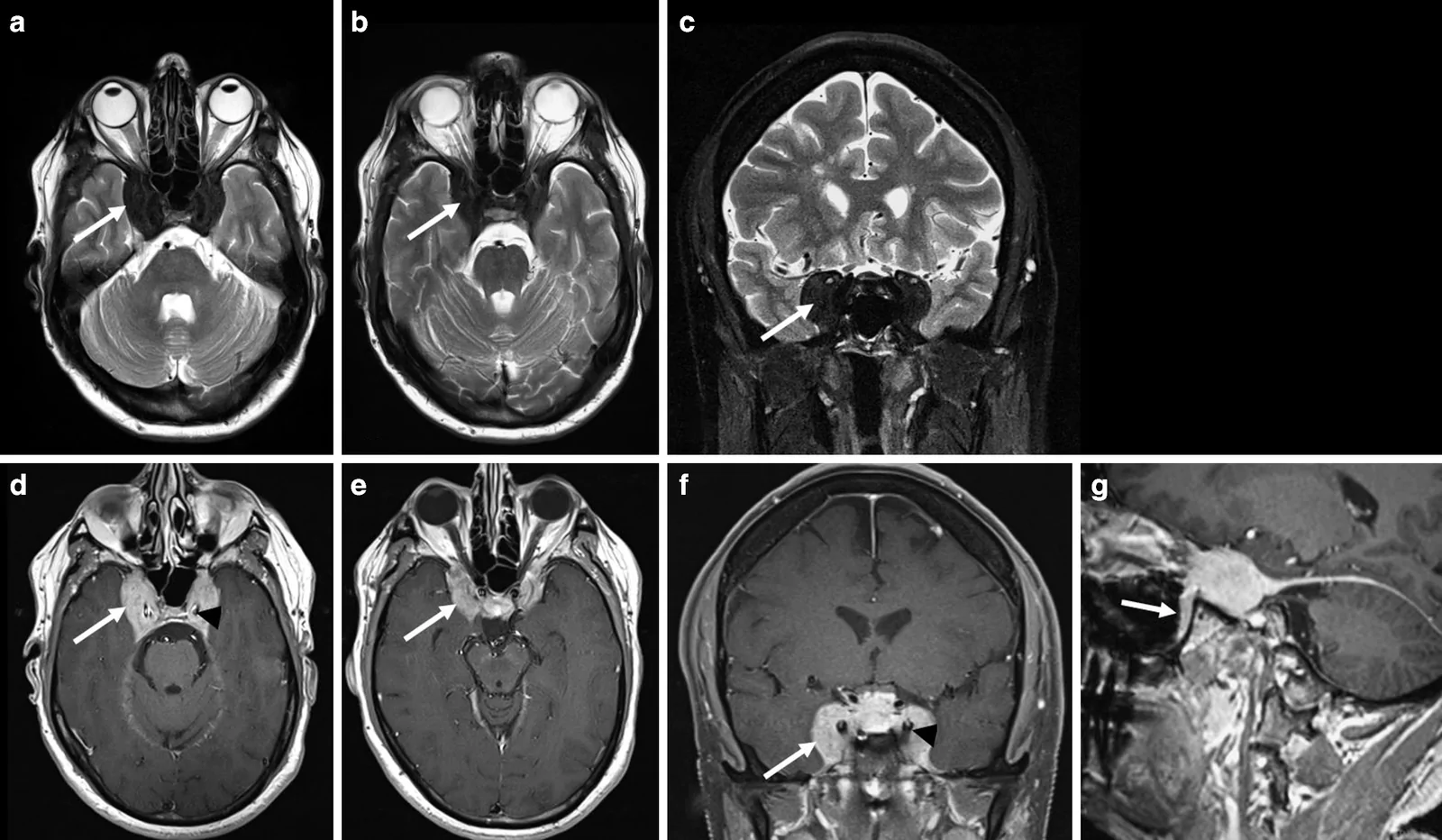
Langerhans cell histiocytosis (LCH), Rosai-Dorfman disease. 63 year old man with slight progressive bilateral abducens nerve palsy (cranial nerve (CN) VI), additional involvement of the left oculomotor nerve (CN III) and the second branch of the CN V (maxillary nerve) bilaterally. **a**–**c** symmetrical hypointense intracavernous space occupying lesion on T2 weighted images (WI; arrow); **d**–**f** distinct contrast enhancement (arrow) encasing internal carotid artery (**d**, **f** arrowhead); spreading along the maxillary nerve (**g** arrow)

Aetiology: According to the WHO classification (2016), LCH is a true neoplastic disease comprising five subtypes [102], i.e. a) LCH (Fig. 19; [102–104]), b) Erdheim-Chester disease [102, 105], c) Rosai-Dorfman disease (Fig. 20; [102]), d) juvenile xanthogranuloma [102, 106], and e) histiocytic sarcoma (Table 1; [102]). It is noteworthy, that the differentiation between LCH and non-LCH is obsolete. CNS involvement in LCH is rare [103]. Imaging pattern: Typical neuroradiological imaging features include granulomatous infiltration of the hypothalamic-pituitary region, often presenting clinically with diabetes insipidus and anterior pituitary insufficiency (Fig. 19, 1; [104, 106]). Neurodegenerative alterations of the brain parenchyma as the second most common manifestation are often associated with symmetrical hyperintense lesions on T2 WI in the cerebellum and basal ganglia (Fig. 1; [103, 105]).

b) Lymphomatoid Granulomatosis

**Fig. 21:**
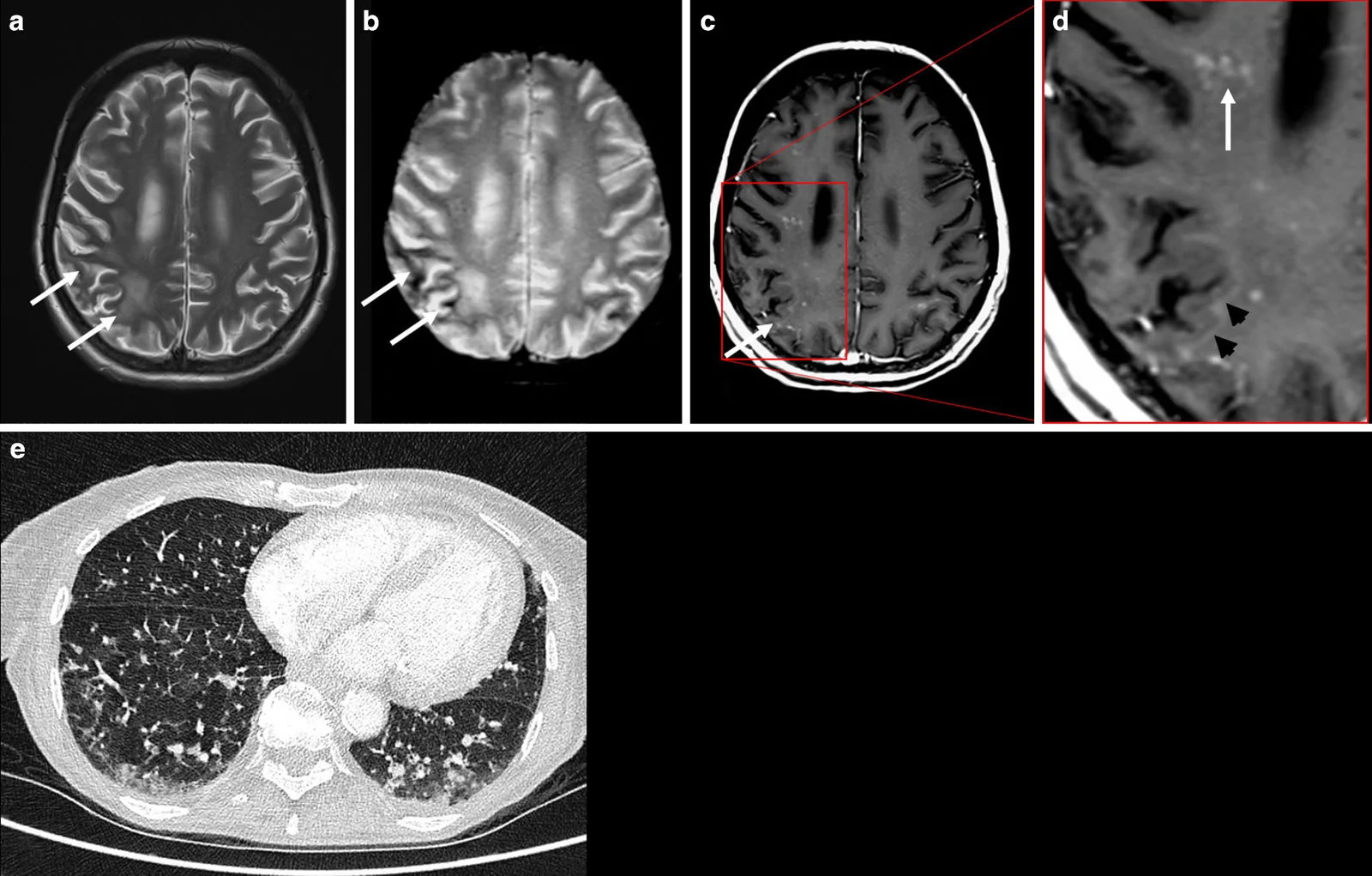
Lymphomatoid granulomatosis. **a**–**d** T2-hyperintense subcortical parietal right-sided lesion (**a** arrow) involving also the U‑fibers with inhomogeneous signal loss on T2*-weighted images (WI; **b** arrow) and multifocal punctuate (**c**, **d** arrow) as well as bandlike subcortical enhancement (**d** arrowhead), note further left-sided parietal lesion; **e** thoracic CT disclosing multiple granulomatous pulmonary lesions

Aetiology: Lymphomatoid granulomatosis is a B-cell lymphoproliferative disorder classified as a subtype of primary CNS lymphoma (PCNSL) [107]. In the context of immunodeficiency, uncontrolled clonal proliferation of latent Epstein-Barr virus (EBV) infected B cells occurs, leading to angioinvasion and angiodestruction [108]. Histopathologically, a triad including nodular lymphoid infiltrates, angiitis with transmural lymphocytic infiltration, and granulomatous changes with central necrosis is observed [107, 108]. Key clinical features: Clinically, pulmonary symptoms predominate, followed by skin manifestations [108]. Additional CNS involvement occurs in approximately one-third of cases and solely CNS affection is rare [107]. Imaging pattern: MRI often demonstrates a perivascular distribution pattern with small, sometimes confluent contrast enhancing lesions and multifocal streaky or nodular T2-hyperintense signal alterations (Fig. 21; [109, 110]). Differential diagnosis: Broad differential diagnoses include especially vasculitides [1, 2], B‑cell lymphomas [107], angiocentric T‑cell non Hodgkin lymphomas [107], sarcoidosis [19, 20, 24], CLIPPERS (chronic lymphocytic inflammation with pontine perivascular enhancement responsive to steroids) [111, 112] and Behçet’s disease [113–115]. Miliary dissemination patterns seen in infectious or malignant diseases must also be considered [85, 86, 107].

## Foreign Body Associated Granulomas

**Fig. 22:**
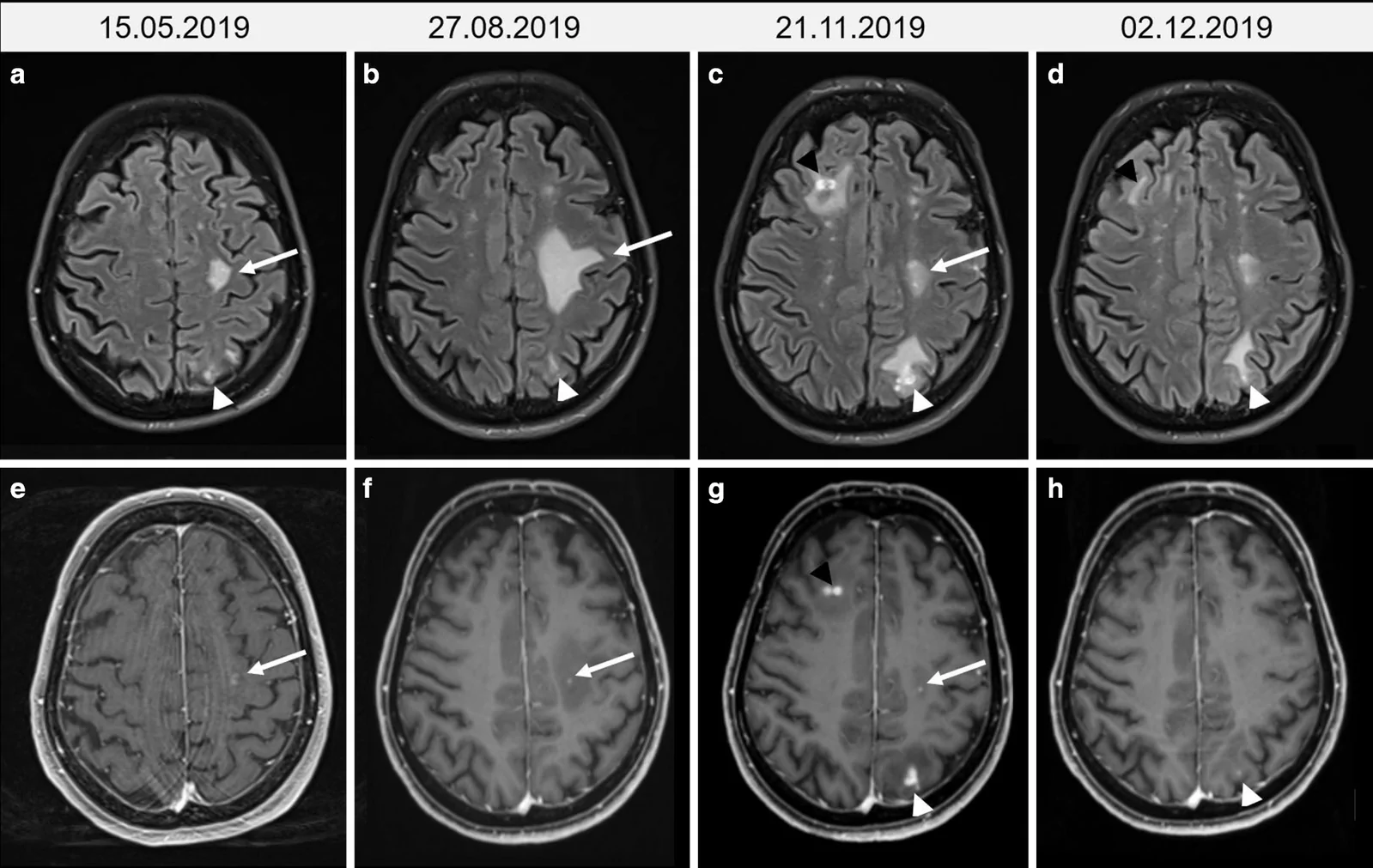
Non ischemic cerebral enhancing (NICE) lesions. **a**–**h** initial routine follow-up MRI in a 59-year old woman several months after coil embolization of an anterior communicating artery aneurysm without additional placement of a stent or flow diverter showing several left-sided hyperintense lesions (**a** arrow, arrowhead; fluid attenuated inversion recovery (FLAIR)), the precentral one with slight enhancement (**e** arrow); follow-up examination 15 weeks later (**b**, **f**) disclosed progressive perifocal signal changes (**b**, **f** ; arrow, arrowhead); **c**, **g** further deterioration left-sided parietal (**c**, **g** white arrowhead) and new right-sided frontal lesions (**c**, **g** black arrowhead), regression of the left-sided central lesion (**c**, **g** arrow); **d**, **h** additional regression of the right-sided frontal and left-sided parietal lesions (**d**, **h** white arrowhead; d: black arrowhead). Note that at any time the patient had neurological deficits

Typical foreign body associated granulomatous changes are listed in Table 1. With the increasing frequency of neuroradiological endovascular interventions and the use of various implant devices with differing compositions and manufacturing processes, procedure-related cerebral foreign body emboli have gained importance [116]. These may result in symptomatic or asymptomatic delayed, non-ischaemic, blood-brain barrier disrupting lesions on MRI, referred to as non-ischaemic cerebral enhancing lesions (NICE lesions) (Fig. 22; [117–119]). The incidence of this rare complication following endovascular procedures ranges from 0.05% to 2.3% [117, 118]. Novel polymer-coated flow diverters appear to be particularly associated with an increased risk in certain product lines [117, 118].

## Other Causes

Granulomatous changes may also occur in the context of immunodeficiency disorders, such as common variable immunodeficiency, or as part of septic granulomatosis [1–3]. Furthermore, drug-induced sarcoid-like reactions have been described, particularly during anticytokine therapy in rheumatology and immune checkpoint inhibitor therapy in oncology (Table 1; [3, 26, 27]). However, a detailed discussion of the various therapeutic agents and associated immunological mechanisms is beyond the scope of this article; readers are referred to the relevant literature.

## Imaging Limitations

Although MRI plays a central role in the differential diagnostic work-up of granulomatous diseases of the CNS, its limitations must be taken into account, particularly considering its low specificity. As shown in Fig. 1 regarding the top differential diagnoses for individual imaging patterns, there is significant overlap in imaging findings between infectious, neoplastic and autoimmune-associated diseases. Thus, circumscribed nodular contrast-enhancing lesions in the subarachnoid space, leptomeninges or parenchyma may occur not only in pathogen-associated inflammation, e.g., neurotuberculosis or in autoimmune-mediated diseases, e.g., PACNS. Also foreign body-associated conditions, e.g., NICE lesions and also neoplastic diseases must be taken into account. Moreover, marked dural involvement may be caused by pathogens in the context of meningeal infection, by autoimmune diseases, e.g., IgG4-RD, as well as by neoplastic conditions, e.g., meningioma en plaque, or even hydrostatic conditions, e.g., SIH.

In this respect, it is essential that, in addition to the seemingly characteristic imaging patterns, both the paraclinical findings—particularly regarding CSF and laboratory analyses—and the clinical course, e.g., acute or slowly progressive, are assessed carefully before a diagnostic classification is made.

In conclusion, aetiology of granulomatous CNS diseases encompass autoinflammatory, autoimmune, infectious, proliferative-neoplastic, foreign body associated, or drug-induced causes. Knowledge of specific imaging patterns is crucial in order to narrow down the broad differential diagnosis and enable timely diagnosis and treatment. Although multiparametric MRI cannot replace histology, its importance in initiating therapy as early as possible is indisputable. This is particularly true since many granulomatous diseases respond to treatment, usually with immunosuppressants and/or anti-infectives. However, it should be noted that the overlap in imaging patterns between infectious, neoplastic and autoimmune-inflammatory diseases results in a partially low specificity. Therefore, additional critical evaluation of both clinical-neurological and laboratory findings, including CSF, is essential for diagnostic classification.

## Data Availability

No datasets were generated or analysed during the current study.
